# Development of novel ALOX15 inhibitors combining dual machine learning filtering and fragment substitution optimisation approaches, molecular docking and dynamic simulation methods

**DOI:** 10.1080/14756366.2024.2301756

**Published:** 2024-01-12

**Authors:** Yinglin Liao, Peng Cao, Lianxiang Luo

**Affiliations:** a The First Clinical College, Guangdong Medical University, Zhanjiang, China; b Department of Pharmacy, Union Hospital, Tongji Medical College, Huazhong University of Science and Technology, Wuhan, China; c The Marine Biomedical Research Institute, Guangdong Medical University, Zhanjiang, China; d The Marine Biomedical Research Institute of Guangdong Zhanjiang, Zhanjiang, China

**Keywords:** Ferroptosis, ALOX15, virtual screening, machine learning, molecular dynamic simulation

## Abstract

The oxidation of unsaturated lipids, facilitated by the enzyme Arachidonic acid 15-lipoxygenase (ALOX15), is an essential element in the development of ferroptosis. This study combined a dual-score exclusion strategy with high-throughput virtual screening, naive Bayesian and recursive partitioning machine learning models, the already established ALOX15 inhibitor i472, and a docking-based fragment substitution optimisation approach to identify potential ALOX15 inhibitors, ultimately leading to the discovery of three FDA-approved drugs that demonstrate optimal inhibitory potential against ALOX15. Through fragment substitution-based optimisation, seven new inhibitor structures have been developed. To evaluate their practicality, ADMET predictions and molecular dynamics simulations were performed. In conclusion, the compounds found in this study provide a novel approach to combat conditions related to ferroptosis-related injury by inhibiting ALOX15.

## Introduction

Ferroptosis is a type of cell death discovered by Dixon et al. in 2012^1^. In contrast to other forms of cell death, such as apoptosis, necrosis and pyroptosis, ferroptosis is characterised by an increase in intracellular peroxides and reactive oxygen species (ROS)^2^. Recent research conducted by Sean K. Ryan et al. revealed that ferroptosis in neuronal cells can cause changes in the transcriptional state of microglia, leading to the development of neurological conditions such as Parkinson’s and multiple sclerosis^3^^,^^4^. In a study by Fang et al. ferroptosis was found to be a key triggering mechanism for ischemia-reperfusion injury-induced cardiomyopath^5^. ALOX family proteins are located in the cytoplasm and are capable of oxidising unsaturated lipids, increasing the amount of ROS and driving the development of ferroptosis^6^. Among them, ALOX5, ALOX12 and ALOX15 are considered to be the most critical enzymes in the process of inducing cellular ferroptosis^7^^,^^8^. In particular, the peroxidation of polyunsaturated fatty acids (PUFAs) catalysed by ALOX15 has been shown to exacerbate ischemia-reperfusion-induced myocardial injury^9^. ALOX15 is also capable of inducing ferroptosis in neuronal cells to exacerbate reperfusion-induced brain injury^10^. According to the X-ray model of the ALOX15 protein published by Gillmor et al. in 1997^11^ and the model of the ALOX15 fatty acid complex obtained by Choi et al. in a further study in 2008^12^, rabbit ALOX15 is a dimer in aqueous solution, and the most stable dimer consists of the two subunits A, B bound. They form a structure containing multiple functional domains: a hydrophobic lipoxygenase active region at the N-terminal end, a linker region in the middle and a phosphorylated region at the C-terminal end. Oxidised fatty acids used as substrates enter the N-terminal hydrophobic pocket of the protein and are catalytically oxidised. Rabbit- and human-derived ALOX15 have a wide range of substrates, mainly free polyunsaturated fatty acids (e.g. linoleic acid, arachidonic acid, eicosapentaenoic acid, and docosahexaenoic acid), but also phospholipids and cholesterol esters in the plasma membrane ^13^^,^^14^. In 2013, Di Venere et al. demonstrated by amino acid residue mutagenesis experiments that the Arg403 residue of human ALOX15B plays a key role in the catalytic oxidation of substrate fatty acids^15^. Intracellular lipid substrates are specifically catalysed to different lipids and phospholipid peroxides according to their atomic spatial arrangement characteristics^16^ and eventually form oxygen radicals, triggering the chain generation of intracellular oxygen radicals. Excessive formation of oxidative free radicals will lead to the rupture of the cell membrane and move the cell towards the death end. Therefore, inhibitors targeting ALOX enzymes have been purposefully developed to find new therapeutic options for injury and degenerative diseases. Despite the low sequence homology (37%) between rabbit and human ALOX15 proteins, their structures are considered to be highly conserved in crystal stacking assays. In particular, in Ivanov et al.’s study, rabbit and human ALOX15 were observed to produce similar movements of their α-18 helices when bound to substrates^17^. Therefore, the conclusions obtained from the above studies based on rabbit-derived ALOX15 may be instructive for the development of human ALOX15 inhibitors, provided that the structure of the human-derived protein has not been fully explored. In 2019, Guo et al. had developed a novel ALOX15 inhibitor, i472, which was able to protect cells from periplasmic polysaccharide-induced cell death and thus inhibit lipid peroxidation^18^. In the following three years, many ALOX15 inhibitory molecules with novel structures were synthesised or discovered. 2020, Ali et al. found that compounds with a 3–(2-naphthyl)-1-phenyl-1H-pyrazole backbone had good ALOX15 binding potential^19^; while Alavi et al. in 2021 discovered a series of compounds in Allylphenols were shown to inhibit the binding of ALOX15 and thus further scavenge oxidative radicals^20^. To date, no ALOX15 inhibitors have progressed beyond the preclinical phase and into the realm of therapeutic agents. This suggests that, although these molecules have demonstrated good target inhibition properties in cytological assays, their complex structures may impede their application in vivo. Consequently, it may be more effective to search for molecules with ALOX15 target inhibition properties from the existing drug library.

Computer-aided drug design (CADD) approaches are more efficient and less costly than traditional experimental screening methods, and thus play a critical role in drug discovery processes^21^. Common CADD techniques involve virtual screening and molecular docking through target structures, yet machine learning and deep learning have been incorporated into the drug discovery process to enhance accuracy^22^. Fragment replacement optimisation is a popular technique for optimising the structure of existing molecules, which involves replacing chemical structures that are not performing well with new ones to create novel molecules with improved characteristics^23^. The replaced fragment and the candidate fragment should be as similar as possible in terms of physical and chemical properties, thereby ensuring that the optimised molecule retains most of the drug-like characteristics of the original lead molecule.

**Figure 1.**
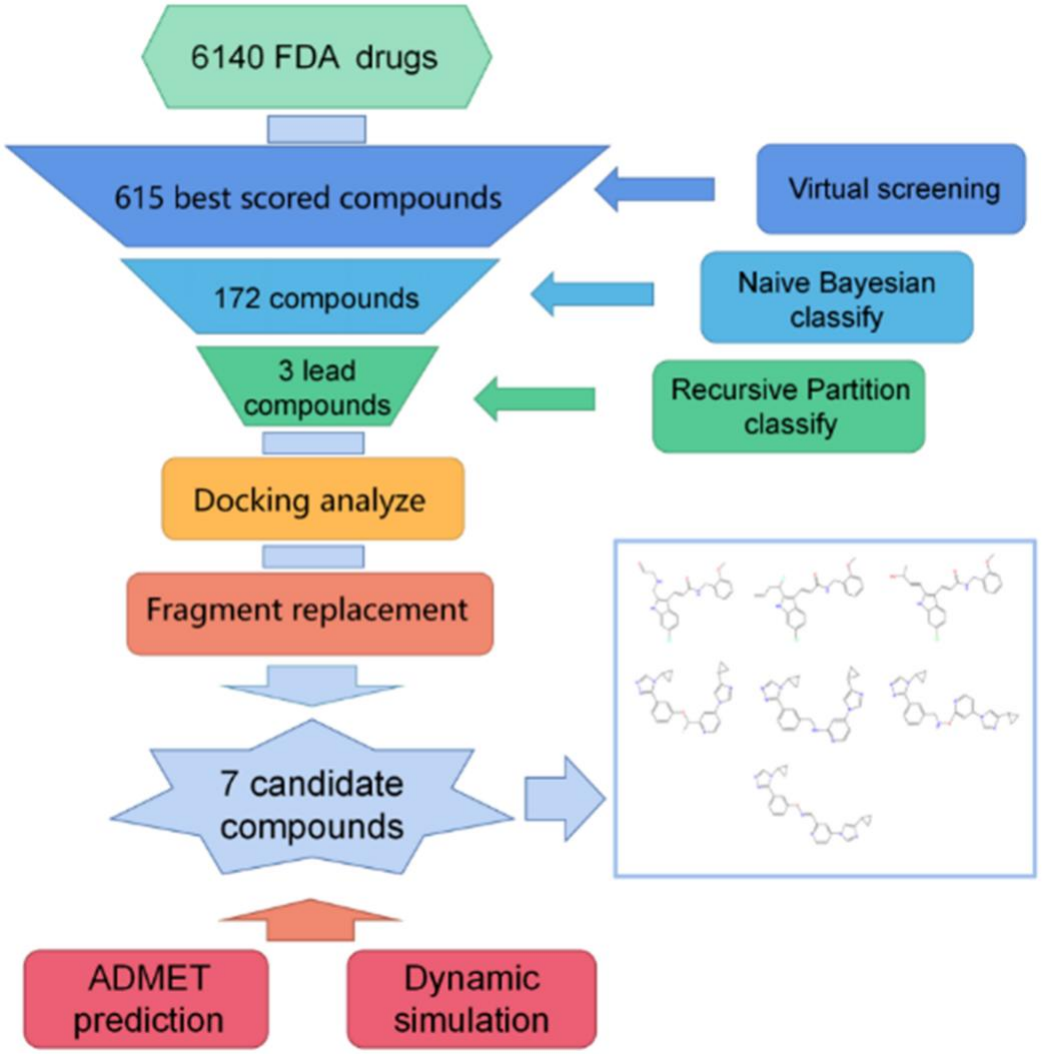
Schematic diagram of the workflow of this study.

In this research, we used structure-based and machine learning virtual screening to pick three compounds from the FDA validated drug library that could bind to the target. Then, by combining these with the previously published ALOX15 inhibitor i472, we identified seven lead compounds with good ALOX15 binding potential through fine docking and fragment substitution modification. ADMET predictions and molecular dynamics simulations showed that these compounds have great potential for in vivo availability. Our workflow is shown in Figure 1.

## Result

### Virtual screening

Libdock module of Discovery Studio was utilised to conduct a virtual screening of 6,140 compounds from the Targetmol FDA compound database, with the Homo sapiens ALOX15 protein model (P16050-LOX15_HUMAN) downloaded from the Alpha fold website as the receptor structure. To validate the docking, the Alpha folded structure was compared to the structure of the PDB rabbit ALOX 15 in superposition, and a known ALOX15 inhibitor was used, as described in the Supplementary Material. When calculating molecular conformations in DS, we used 20 kcal/mol as the energy threshold and set the sp2-sp2 bond within each molecule to be rotatable in order to calculate and take the stable conformation with the lowest energy for docking with the protein. The screening process yielding a total 41,546 available conformations. Ultimately, 2,458 molecules were successfully docked to the ALOX15 structure, with scores ranging from 25.36 − 147.87. In general, a higher Libdock score predicts a better interaction profile. For the FDA drug database used for screening, we also provided the initial characterisation of their Libinski-like drug properties in the Supplementary Material. After the initial Libdock screening, the number of molecules with poor drug similarity in the candidate compound database dropped dramatically from 3,185 (51%) to 102 (4%), which significantly reduced our cost of subsequently excluding non-drug molecules. To further reduce the pool of candidates, we selected the 615 molecules that scored in the top 25% to proceed to the next step of the study.

### Machine learning - QSAR

#### Data set preparation, characterisation and down-scaling

For reasons of article length and readability, we have placed the methods and results of characterisation of molecular properties and data down-scaling within the database in the Supplementary Material.

### QSAR model construction and testing

#### Naive Bayesian classification model

**Figure 2.**
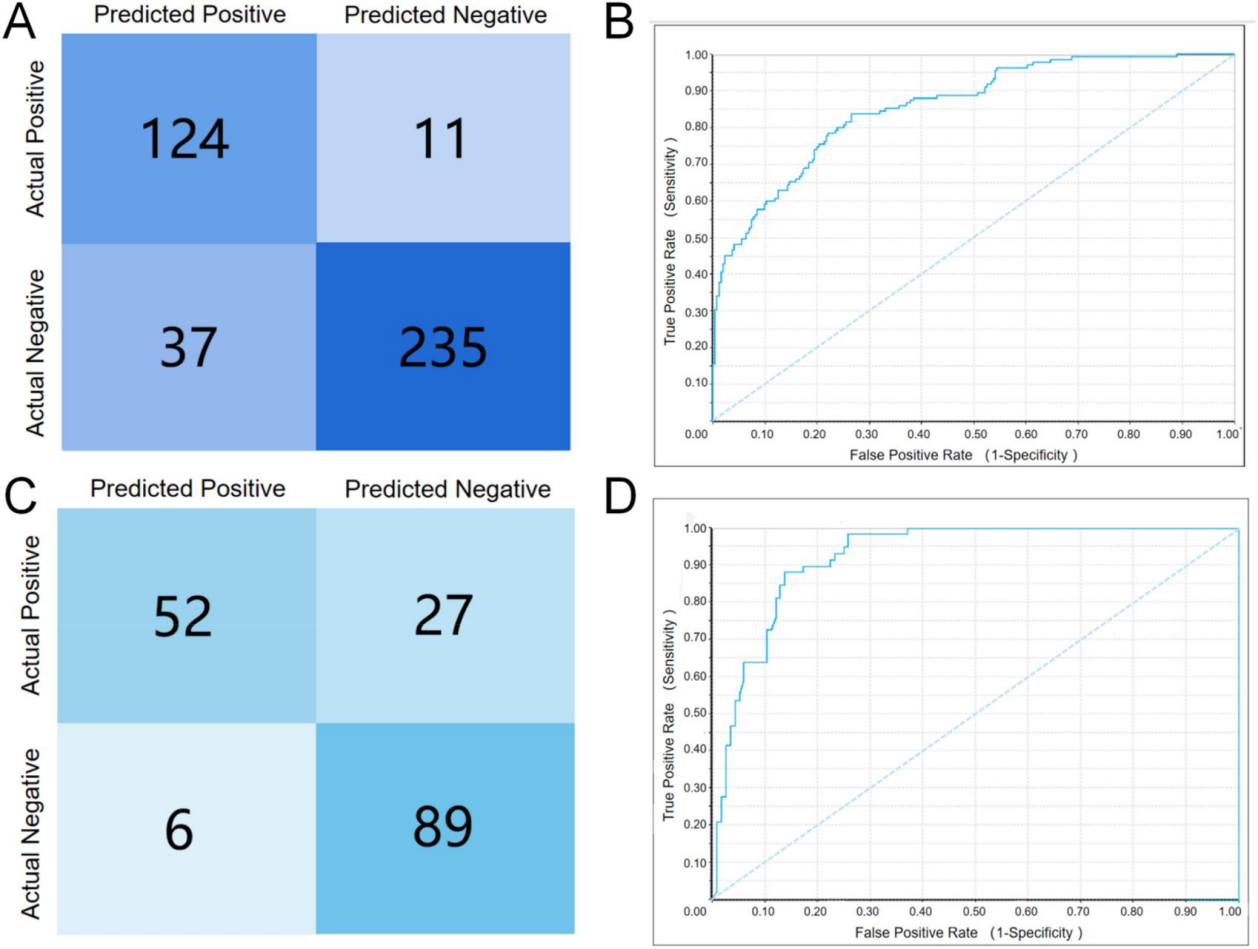
Internal/external validation ROC curves and confusion matrix for the Naive Bayesian model. (A)Confusion matrix for 10-fold validation of the model internal; (B) ROC curve for 10-fold validation of the model internal; (C) Confusion matrix for the internal external test of the model; (D) ROC curve for the external test of the model.

As shown in Table 1, the internal validation based on 10-fold validation showed excellent performance with a true positive recognition rate of 91.90% and a true negative recognition rate of 86.40%. The validation results using external test sets, on the other hand, showed excellent evaluation results despite being somewhat poorer than 10-fold validation. The ROC curves and the predicted-true value confusion matrix for internal and external validation are shown in Figure 2. In both 10-fold validation and external validation results, the model has a higher sensitivity than specificity, meaning that the model is better at identifying active molecules than inactive ones. The Precision value of the model, which represents the percentage of true positives out of the total number of compounds classified as positive, was 0.66 and 0.77 for the internal/external tests respectively, which is worse compared to the sensitivity. This may be explained by the fact that the model identifies false positive examples at a higher rate than false negatives. The reliability of the model was also assessed using both the F-1 score and the MCC metric, and the desired results were obtained. ROC rating is a model quality evaluator based on ROC sore, when the score is greater than 0.75, the model will be evaluated as "Good" in testing. Correspondingly, scores greater than 0.9 will be evaluated as "Excellent". Both in the 10-fold validation and in the test set validation, the Naive Bayesian model shows outstanding Sensitivity and Specificity (both greater than 0.75), and receives "Good" and "Excellent" ratings respectively. Combined with the MCC, F-1 score and other auxiliary indicators, we have sufficient reasons to assume that the NB model has a good ability to discriminate between active and inactive molecules.

**Table 1.** Internal/external validation of the Naive Bayesian model.

Validation	Sensitivity	Specificity	ROC score	ROC rating	Precision	F-1 score	MCC
10-fold validation	0.92	0.86	0.86	Good	0.66	0.77	0.63
External test	0.90	0.77	0.92	Excellent	0.77	0.83	0.75

### Recursive partition forest classification model

**Figure 3.**
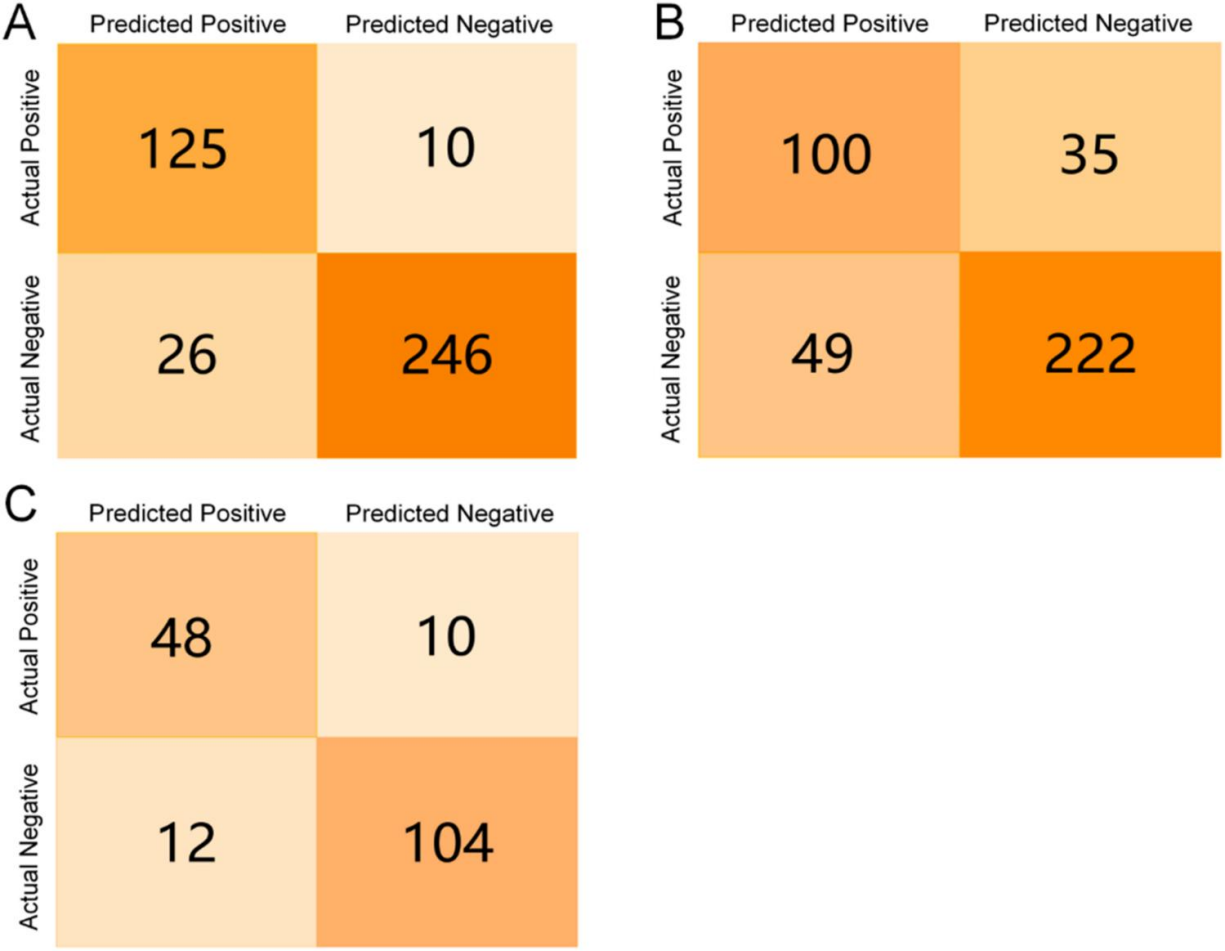
Confusion matrices for in-bag/out-of-bag validation/test set validation of the Recursive Partition model. (A) Confusion matrix for model in-bag validation; (B) Confusion matrix for model internal out-of-bag validation; (C) Confusion matrix for model test set validation.

We measured the quality of the recursive partitioning model by in-bag validation and out-of-bag validation and test set validation, respectively. While samples are played back (in-bag) during model formation using the bagging algorithm, some samples are often missed during the sampling process (about 37%). Since these samples are not used for model training, they are suitable for external testing, referred to here as out-of-bag testing. The test set consisted of 174 ALOX15 inhibitor molecules that we prepared in advance. The results of the model testing are shown in Table 2, and the confusion matrix is displayed in Figure 3. It is generally accepted that higher values of sensitivity, specificity, accuracy and F-1 score in the range of 0.5–1 indicate a better classification ability of the model. As for the MMC value, the higher the score in the range of 0–1, the more reliable the model is. It is clear that the in-bag validation shows excellent results for the model: the sensitivity and specificity are higher than 0.9, and the precision and MCC values indicate a small deviation between the test results and the actual classification. However, in the out-of-bag validation, all evaluation metrics decreased significantly, but within acceptable limits. Additionally, the model demonstrated far better classification performance on the test set than out-of-bag validation, approaching in-bag validation in all performance metrics. In conclusion, although not as good as the results of in-bag validation, the results of out-of-bag validation and test set validation results generally support the hypothesis that recursive partitioning models are capable of classifying data well.

**Table 2.** In-bag/out-of-bag validation of the Recursive Partition model.

Validation	Sensitivity	Specificity	AUC	Precision	F-1 score	MCC
In-bag training	0.93	0.90	0.97	0.83	0.87	0.81
Out-of-bag training	0.74	0.82	0.86	0.67	0.70	0.57

### Dual filter based on both classification models

A combination of both Naive Bayesian model and Recursive Partition Forest model was used for a dual filter. Our aim was to limit the range of positive candidate compounds as much as possible, and since the NB model exhibited a much lower average false positive rate across validations (as shown in Supplementary Table S7), we used it as the first screening method. 172 molecules were classified as "active" by the NB model for the 615 compounds obtained from the previous high-throughput screening. For these 172 molecules, we further classified them using the RP-Forest model and identified three inhibitor molecules, OSI-930, SD-208 and GS-444217, using P(1) > 0.6 as the criterion for inhibitor status, their structures are shown in Table 3.

**Table 3.** Structure, Libdock and CDOCKER energy scores of the three lead molecules and the control inhibitor i472.

Molecule	Structure	Libdock score	CDOCKER ENERGY	CDOCKER INTERACTION ENERGY
OSI-930	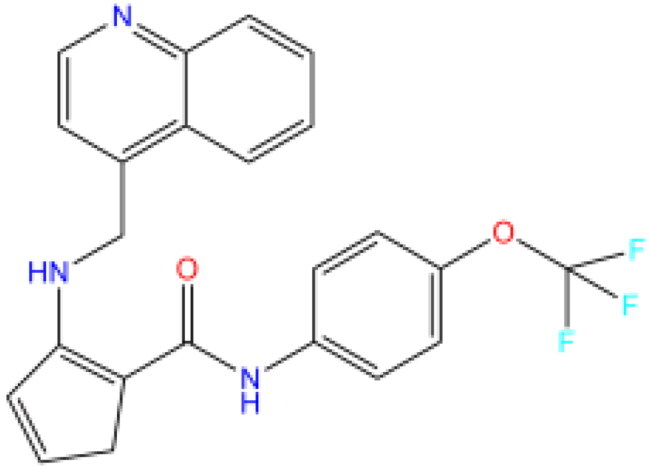	110.36	−17.94	−12.13
SD-208	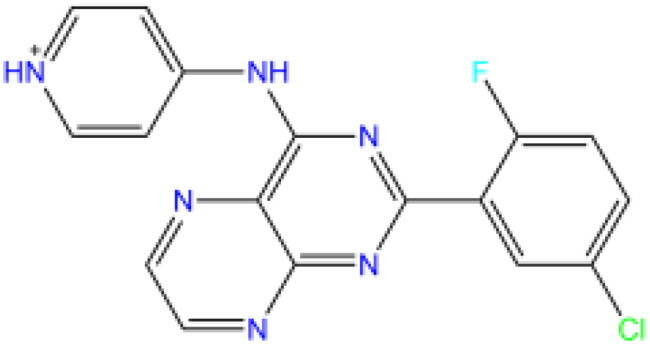	93.16	−23.89	−17.31
GS-444217	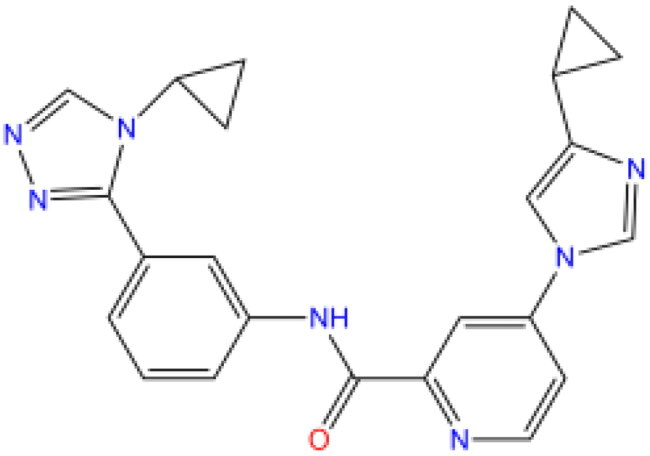	119.96	−33.87	−21.49
i472	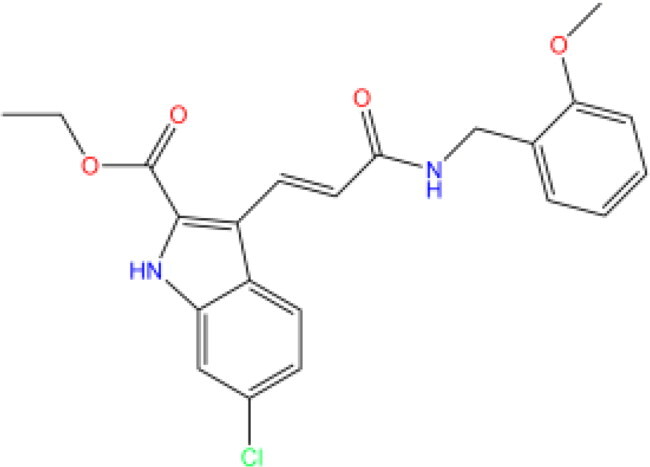	119.59	−34.13	−29.64

To verify the suitability of our screening method, we corrected the external validation confusion matrix for the NB model. We switched the screening order between the NB model and the RP model in our protocol and retested seven candidate molecules. In order to harmonise the prediction criteria of the two models, we considered the molecules with a score greater than 0.5 in the screen as active molecules. The results showed that these seven molecules also passed the RP-NB screen. This result is shown in Supplementary Table S8. For the 615 molecules that were used in the ML screen, 221 passed the RP model and 193 passed the NB model.

### Refine docking analysis

After the first step of high-throughput virtual screening and a double filiter using machine learning QSAR model, we initially obtained three possible ALOX15 inhibitor molecules. To further determine their interactions with the targets, we performed refined docking analysis for these compounds by the CDOCKER module and selected the known inhibitor i472^18^ as a control molecule. Libdock, CDOCKER docking energy scores of all ligands are shown in Table 3, and their interaction with the protein is presented in Supplementary Figure S5.

We have analysed binding status of the three molecules to their targets in terms of both ligand-target binding interactions and docking energy scores. Docking energy scores consist of CDOCKER ENERGY, which represents the pure binding strength between ligand and target, and CDOCKER INTERACTION ENERGY, which additionally calculates spatial collision and potential energy on top of the latter.

OSI-930 forms seven (out of a total of 12) interactions with ALOX15, of which Pi interactions make up the larger part, with the molecule only forming hydrogen bonds and Halogen interactions with GLU536 and ARG402 residues, respectively. For the other two molecules, SD-208 and GS-444217, their interactions with the targets are also dominated by Pi interactions: the former formed 18 interactions with the target, including only one carbon-hydrogen bond with GLU356 and one Halogen bond with ILE662 residue; the latter formed 14 interactions with the target, except for alkyl and carbon-hydrogen bonds with PHE352 and GLY406, all of which were Pi interactions. bond with PHE352 and GLY406 were all Pi interactions. Given that Pi interactions are mostly formed between aromatic or five-member rings and residues of small molecules, we believe that adding some degree of aromatic ring number is critical to enhance the molecule’s ability to bind to the ALOX15 target. Notably, there is an unfavourable bump between GS-444217 and residues GLU356, LEU407 and ILE413 of the target. 2020, Cruz et al. demonstrated that residue 596 plays a key role in maintaining the structural stability of ALOX15^24^. In contrast, three molecules obtained after a dual screening by docking virtual screening and machine learning models, as well as the control inhibitor i472, all formed stable non-covalent interaction interactions with this residue, confirming the ALOX15 inhibition potential of these lead compounds.

### Lead compound fragment substitution

#### Optimisation of the fragment replacement of the control compound i472

In order to maximise the target inhibition efficiency of the molecule with less impact on the original properties of the inhibitor, we optimised their structure using a fragment with high physical-chemical similarity to the original fragment structure and with higher polarity. From the results of the exact docking analysis in the previous step, the ester-based fragment of the ALOX15 inhibitor i472 that did not interact with the target was replaced. 96 novel molecules were obtained by fragment search substitution. After Libdock docking screening, 72 molecules were obtained that successfully docked to the ALOX15 structure. Among them, five novel compounds with higher docking scores than the control compound i472 and more favourable interactions were further selected and named i472a-i472e. i472’s original and optimised structures, docking scores are shown in Table 4. Interactions of i472a-i472e with the target are shown in Supplementary Figure S6.

**Table 4.** Novel molecules obtained by i472 fragment replacement optimisation.

Molecule	Structure	Libdock score	CDOCKER ENERGY	CDOCKER INTERACTION ENERGY	Number of favourable interactions	Average interaction distance
i472	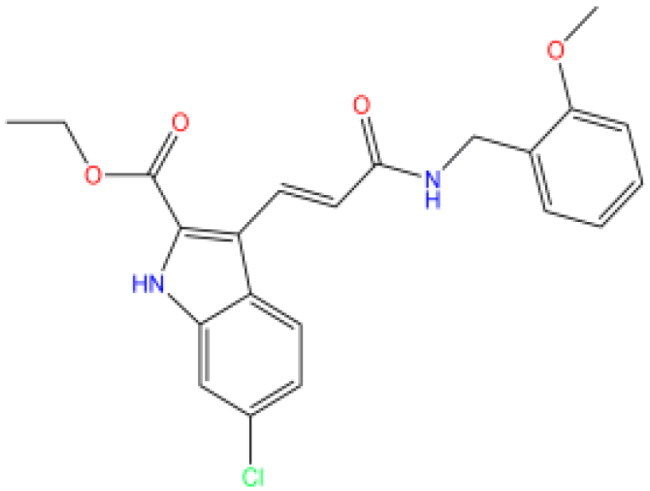	119.59	−34.13	−29.64	14	3.59 Å
i472a	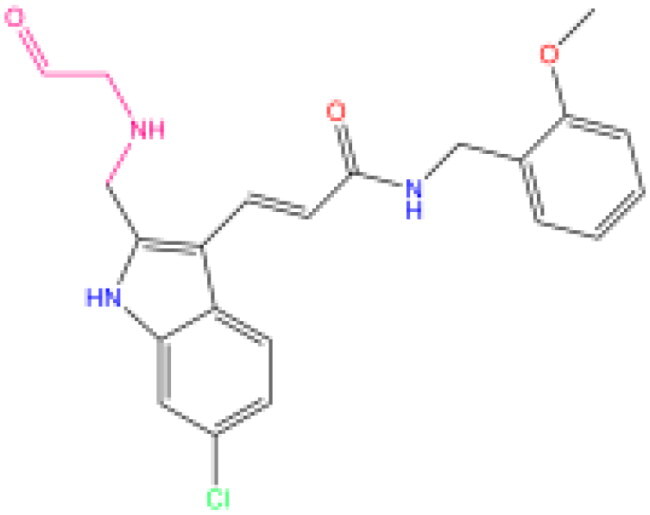	130.67	−44.09	−32.58	15	3.34 Å
i472b	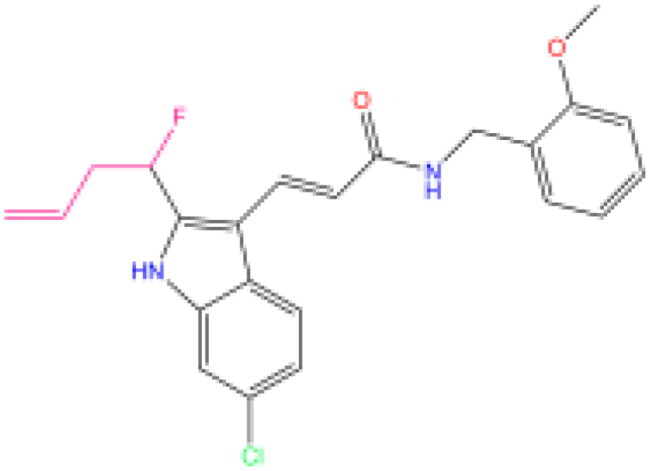	125.91	−37.14	−31.08	15	3.37 Å
i472c	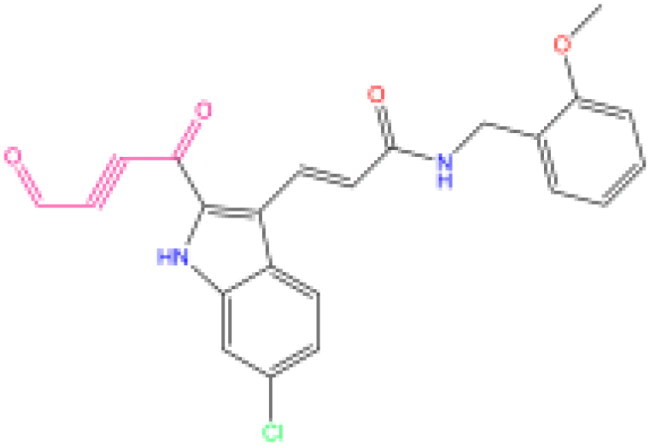	123.90	−32.02	−29.15	14	3.24 Å
i472d	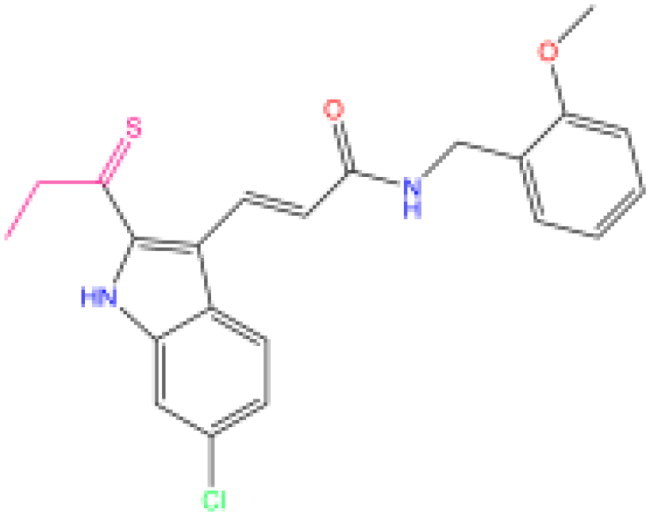	122.04	−36.15	−33.88	13	3.45 Å
i472e	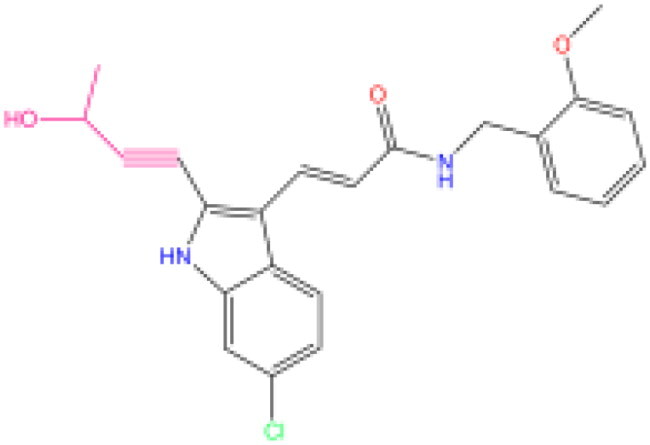	121.54	−35.06	−28.90	16	3.38 Å

Comparing the new molecules i472a-e obtained by substitution from the original structure i472, it is readily apparent that the carbon-hydrogen bond formed by the four novel molecules and ILE662 disappears, except for i472c. In this regard we speculate that this may be because the new substituted fragment forms an additional interaction with the acceptor, bringing the substituted end of the molecule closer to the residue and correspondingly further away from the acceptor residue at the other end (see Table 4). On the other hand, the carbon-hydrogen bond is more dependent on the proximity of the molecules relative to the Pi interaction, which explains the change in the receptor interaction of i472a-e compared to the original structure. i472a forms an additional carbon-hydrogen bond with the receptor GLY406 and GLN416 (distance of 2.37 Å) and hydrogen bond (at a distance of 2.33 Å), while i472 only forms van der Waals force interactions with these two residues. In contrast, the other end of the molecule has a reduced hydrogen bond with the acceptor residue LEU407. i472b and i472d have similar interactions with the acceptor, with the olefin structure at the end of the former’s new substituted fragment forming an additional Alkyl interaction with LEU182 and LEU588 respectively; the latter’s alkyl substituted fragment also forms an additional Alkyl interactions. For i472c, the substituted fragment only forms an additional hydrogen bonding interaction with the residue, but at a much closer distance (2.37 Å). It can be observed that the carbon-hydrogen bond between the other end of the molecule and ILE662 is still retained, and an additional Pi interaction is formed with ARG402. This is probably due to the stronger carbon-hydrogen interaction with residue GLY406 (2.98 Å) in the mid-molecular structure. i472e forms a hydrogen bond (2.36 Å) and a carbon hydrogen bond (2.80 Å) with the substituted fragments GLY406 and GLY412, which is similar to that of i472a. In general, the substituted part of the original structure of i472 is an ester group, which is less likely to interact with other residues due to its lower polarity. The two ideas for optimisation of i472 that have emerged from our work so far would be: firstly, to use a terminal fragment with an alkyl group for substitution, which could lead to additional Alkyl interactions of the molecule with LEU182, as observed in the interactions of i472b and i472d with ALOX15; secondly, to use a terminal fragment that can easily form a hydrogen bond to the ester group for substitution, the principle being to increase the polarity of the structural polarity of the branching. The five newly generated molecular structures do not differ significantly from the original inhibitor molecules in terms of backbone, with substitution only in the fragments of the side branches, but it is also observed that these molecules behave better than the original structures in terms of binding energy and interaction level.

#### Optimisation of fragment replacement of candidate lead molecules

In order to explore the possible scope for structural improvement of the three lead molecules, we modified them by fragment substitution based on the results of fine docking analysis. Considering that SD-208 forms many interactions with the receptor, we believe that it already has good lead potential by itself. On the other hand, the branched chains of SD-208 structure are all aromatic ring structures, which lack sufficient room for modification, so we only modified OSI-930 and GS-444217 by fragment substitution.

#### OSI-930

Based on the results of precise docking (seen Supplementary Figure S7), we found that the monocyclic phenylamino structure of OSI-930 near residues ALA403-PHE414 of the receptor is less efficient in forming interactions with the receptor compared to the other parts of the binary/five-membered ring. For this reason, we substituted this part of the fragment, generating 298 new molecules. After Libdock screening, six molecules were obtained that successfully docked with ALOX15, osia-osif. Their structures and docking scores are shown in Table 5, where we found that osia had a superior docking score than the original structure of osib. However, although the docking scores of the other molecules were not as high as the original structure of OSI-930, they still formed more interactions with the target than they otherwise would have. Taking into account the docking energy score and the number of interactions, we finally selected osia as the best lead in this set of optimised molecules.

**Table 5.** Structural and target interaction information for OSI-930 and its fragment replacement optimised molecule osia - osif.

Molecule	Structure	Libdock score	CDOCKER ENERGY	CDOCKER INTERACTION ENERGY	Number of favourable interactions	Average interaction distance
OSI-930	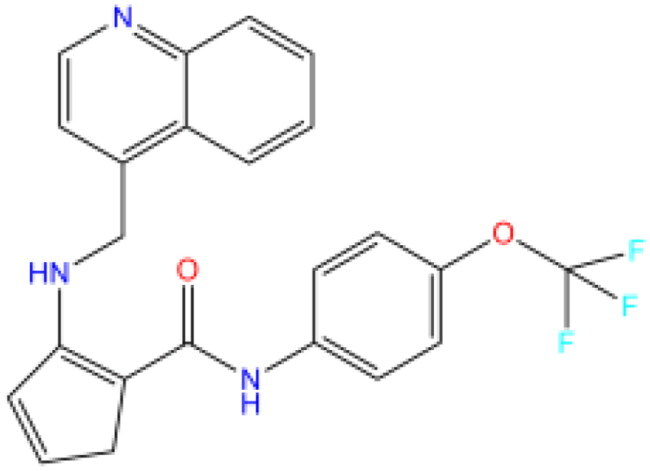	110.36	−17.94	−12.14	12	3.30 Å
osia	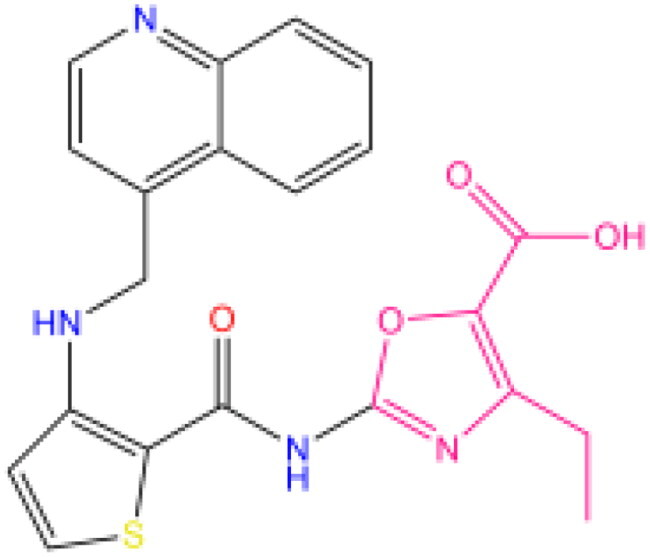	121.72	−20.40	−14.70	17	3.30 Å
osib	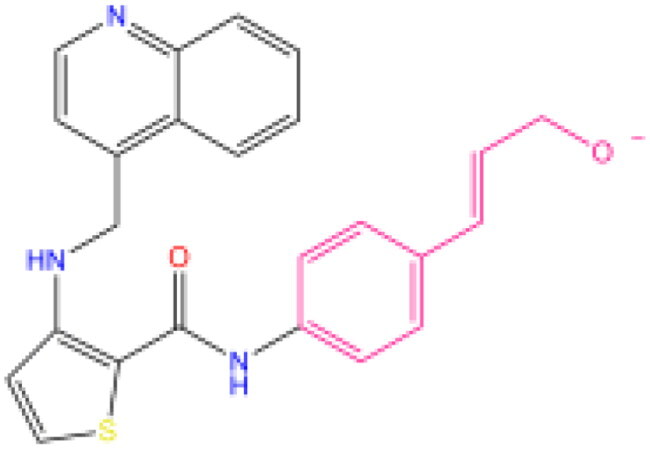	106.49	−15.74	−13.63	13	3.39 Å
osic	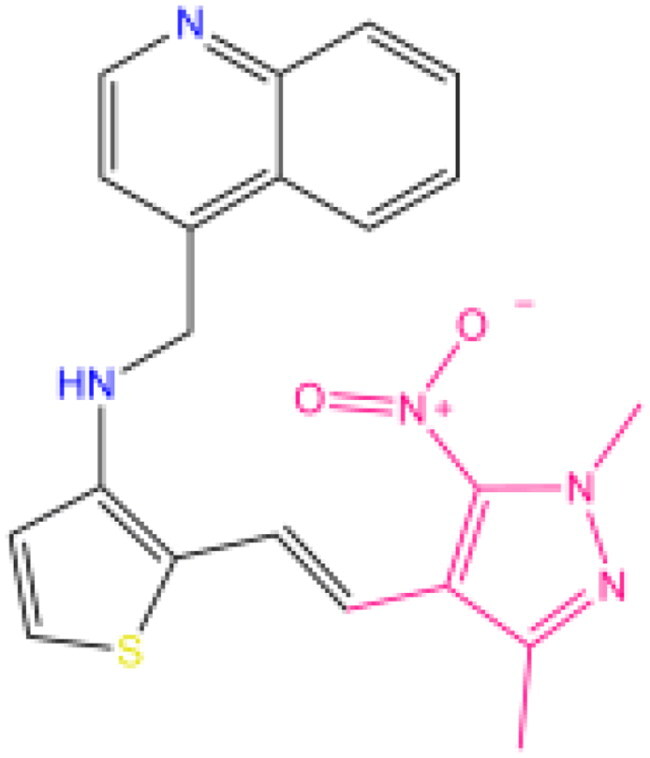	105.46	−19.97	−15.59	24	3.37 Å
osid	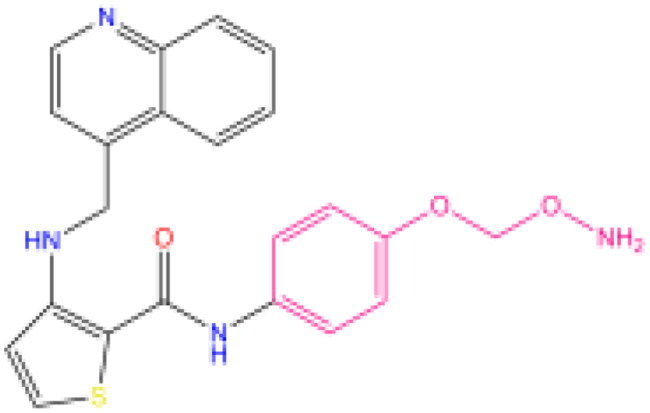	97.80	−15.04	−9.53	14	3.22 Å
osie	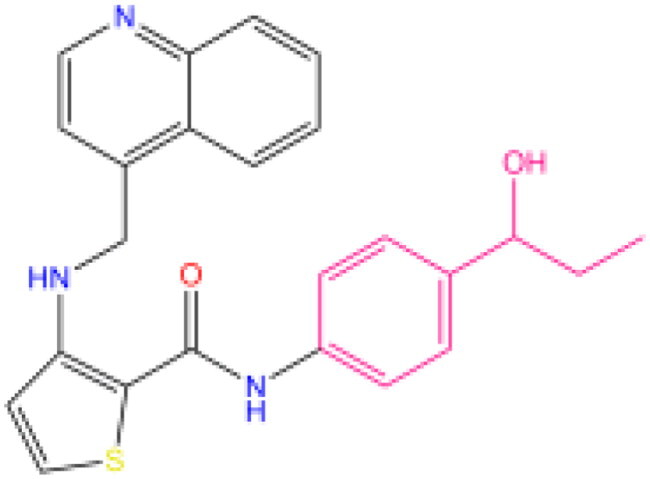	90.13	−13.32	−10.81	14	3.24 Å
osif	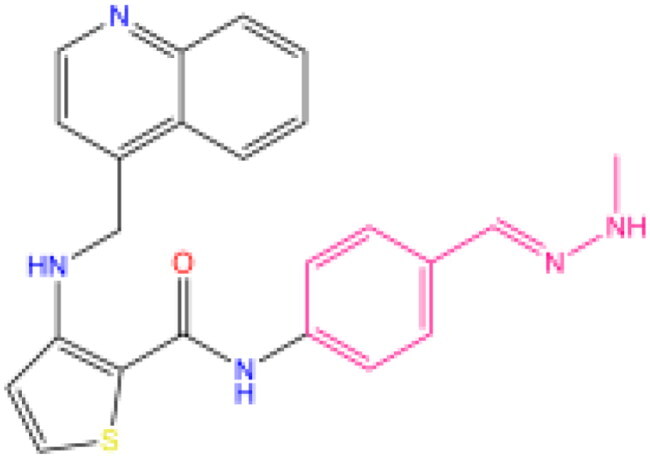	70.25	−14.59	−11.08	13	3.24 Å

#### GS-444217

The results of refined docking showed that there were more unfavourable collisions between GS-444217 and the receptor (seen Supplementary Figure S8). Compared to the other two candidate lead compounds, OSI-930 and SD-208, we found that GS-444217 has a significantly longer structure and is more likely to collide with narrow pockets when the molecule is not sufficiently soft in conformation. The molecule relies primarily on the ring structure to form interactions with the receptor residues, whereas the long linker between the ring groups is a stable amino-peptide bond structure that is less prone to bending and largely non-interactive with the receptor. We therefore considered substitution of linker fragments to increase their polarity/reactivity or their target binding ability. The substitution generated 38 novel ligands, of which eight molecules had better docking scores than the GS-444217 structure for the molecule gsa–gsh, as shown in Table 6. All of the newly generated molecules, gsa–gsh, had the same or higher number of interactions than the original structure GS-444217.

**Table 6.** Structural and target interaction information for GS-444217 and its fragment substitution optimised molecule gsa–gsh.

Molecule	Structure	Libdock score	CDOCKER ENERGY	CDOCKER INTERACTION ENERGY	Number of favourable interactions	Average interaction distance
GS-444217	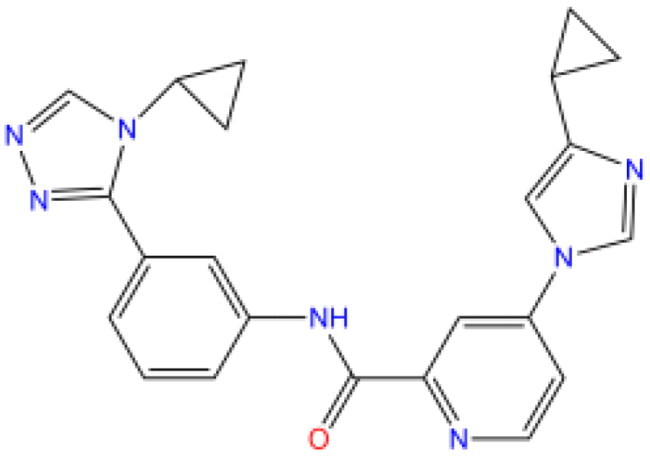	119.96	−33.87	−21.49	14	3.47 Å
gsa	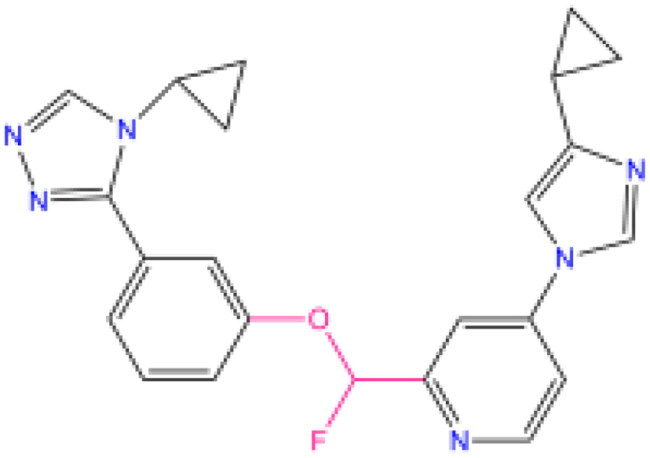	148.47	−54.32	−29.64	22	3.42 Å
gsb	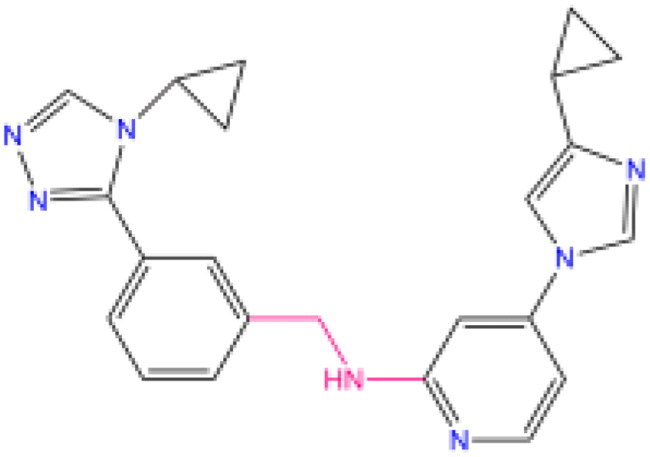	134.33	−34.37	−17.56	17	3.48 Å
gsc	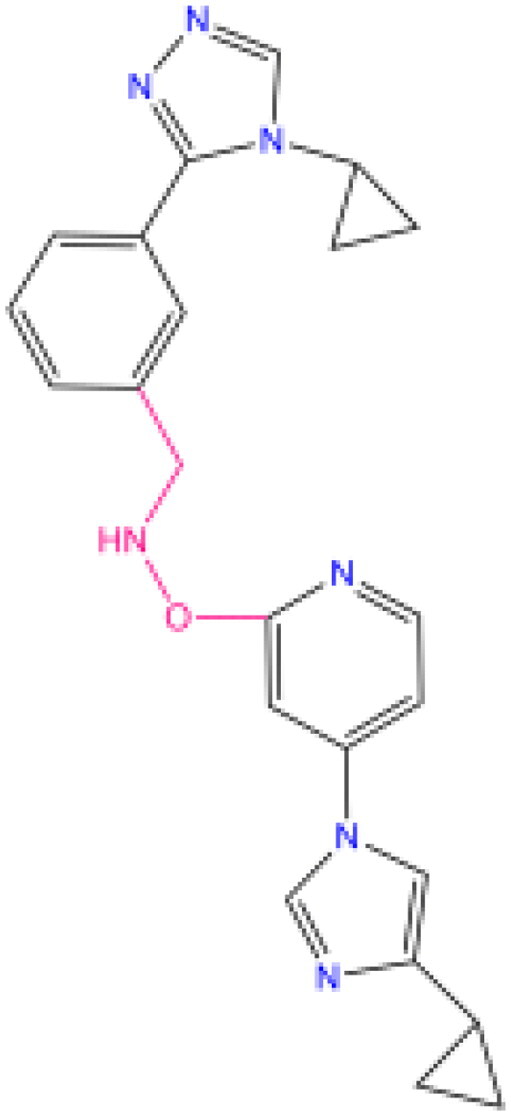	129.87	−47.85	−24.82	20	3.43 Å
gsd	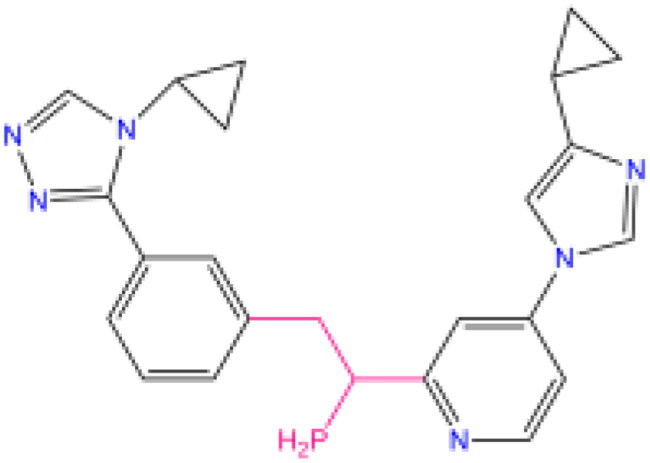	129.78	−31.20	−18.65	14	3.55 Å
gse	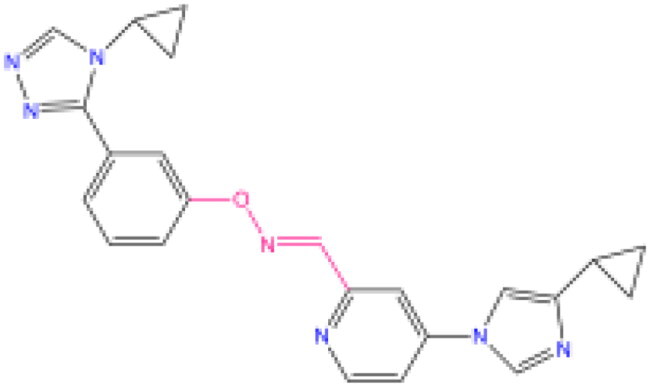	129.08	−35.76	−18.47	19	3.41 Å
gsf	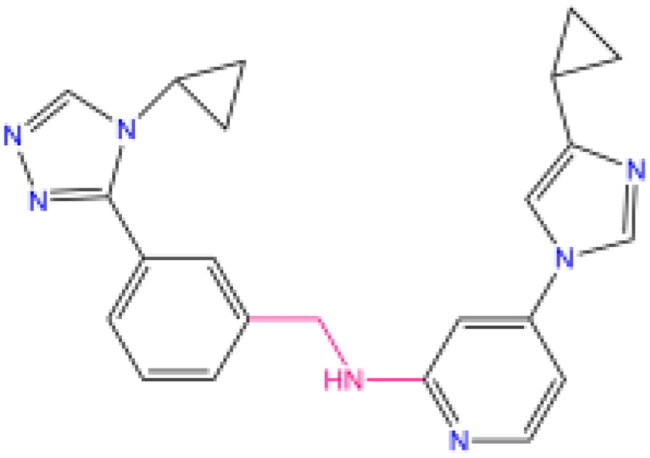	125.36	−32.88	−27.89	23	3.48 Å
gsg	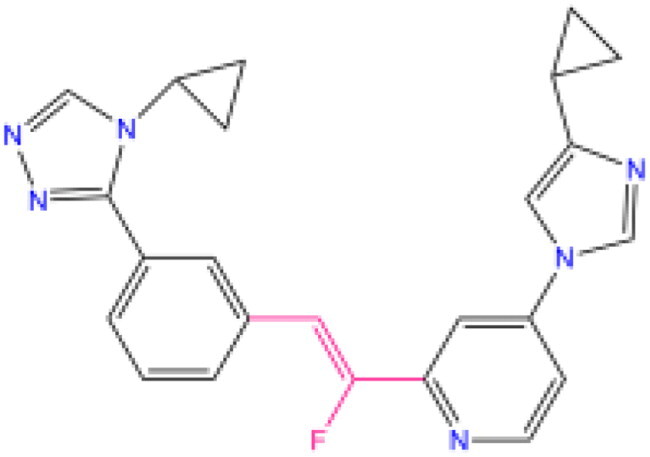	123.37	−33.89	−20.11	17	3.23 Å
gsh	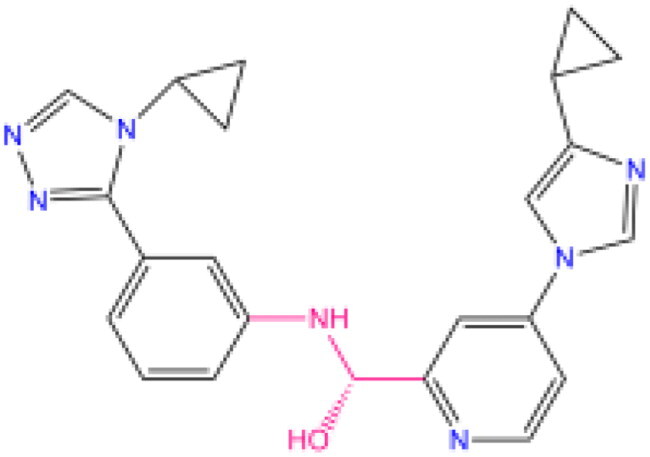	120.52	−27.11	−19.99	17	4.21 Å

**Figure 4.**
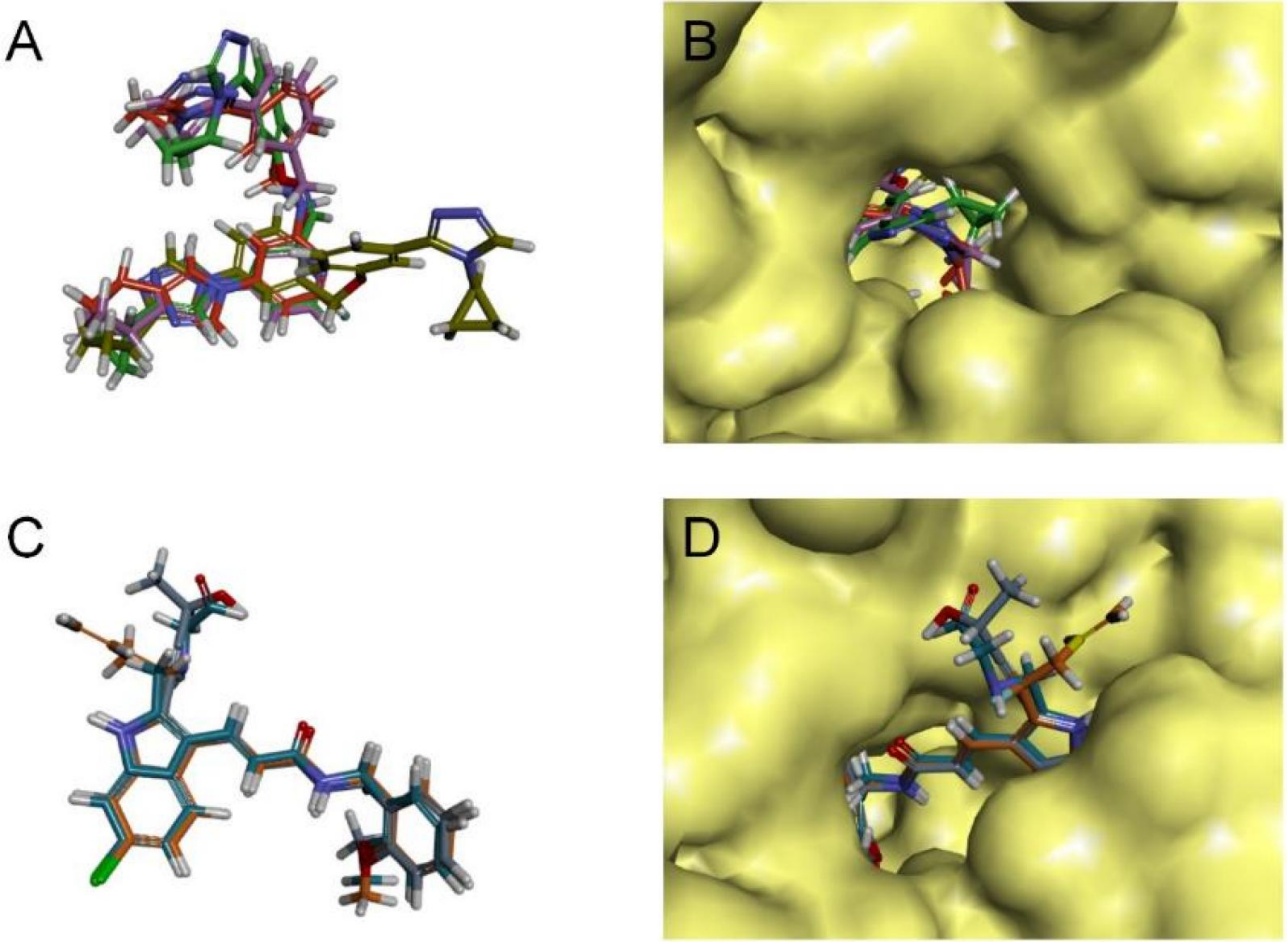
Structural superposition schematic of candidate compounds. (A) Structural overlay of gsa/b/c/e, gsa is earthy yellow, gsb is red, gsc is purple, and gse is green; (B) Schematic representation of the binding of gsa/b/c/e to the active pocket of ALOX15 protein; (C) Structural overlay of i472a/b/e, i472a is cyan, i472 is orange, and i472e is grey; (D) Schematic representation of the binding of i472a/b/e to the active pocket of ALOX15 protein.

**Figure 5.**
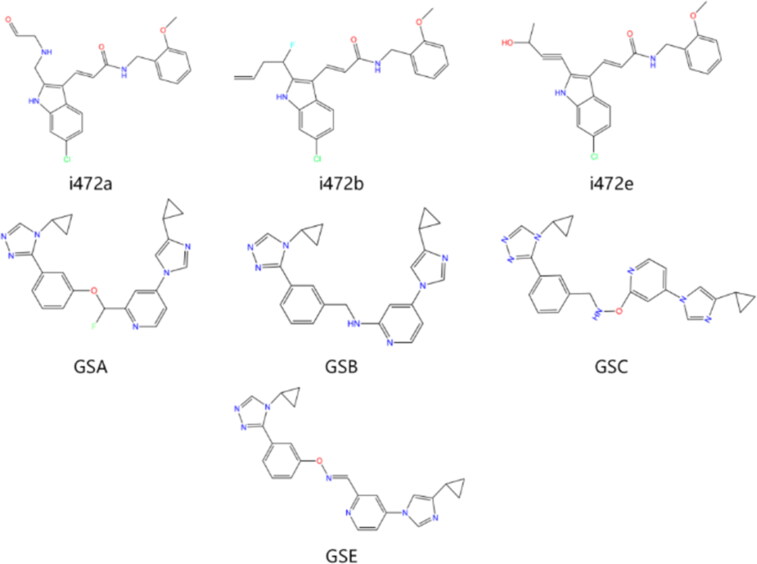
Final selection of the lead compound structure.

Combining all the above screening and molecular structure optimisation steps, we obtained a total of 26 novel molecules that were considered to have good potential for ALOX15 target inhibition, namely the FDA drugs OSI-930, SD-208 and GS-444217 and their derivatives. However, in order to select the most representative inhibitor molecules, we retained the compounds that outperformed the control inhibitor i472 in terms of Libdock score, CDOCKER ENERGY, and number of favourable interactions as the lead compounds for final consideration: the fragment - optimised compounds gsa, gsb, gsc, gse and ia, ib, i.e. they all integrated tightly into the catalytic pocket of ALOX15 (Figure 4). Their chemical structures are shown in Figure 5.

### ADMET property studies

To confirm the actual druggable properties of the lead molecules obtained from the above steps, we used the online tool SwissADME to predict the in vivo absorption, in vivo distribution, metabolism, excretion and toxicity properties of the seven lead compounds. Specifically, we discussed the lipid-water distribution coefficient, blood-brain barrier permeability, gastrointestinal absorption, P-gp drug pump protein inhibition and hepatic drug enzyme inhibition, and drug-likeness of the seven compounds, with the specific values shown in Table 7. All molecules were predicted to have good gastrointestinal absorption, which indicates their excellent potential for oral utilisation in practical medicinal use.

**Table 7.** ADME test results for the seven lead molecules.

Molecule	Molecule Weight (g/mol)	Log *P*_o/w_ (iLOGP)	Solubility	BBB Permeant	Number of Hydrogen Bond Acceptor	Number of Hydrogen Bond Donor Number	Number of rotatable bond	GI absorption	CYP2D6 inhibition	P-gp substrate
i472a	411.86	2.93	Soluble	No	4	3	10	High	Yes	Yes
i472b	412.88	3.61	Moderately soluble	Yes	3	2	9	High	Yes	No
i472e	408.88	3.54	Moderately soluble	Yes	3	3	6	High	Yes	No
gsa	416.45	3.35	Moderately soluble	Yes	6	0	7	High	Yes	Yes
gsb	397.48	2.83	Moderately soluble	Yes	4	1	7	High	Yes	Yes
gsc	413.48	3.30	Soluble	No	6	1	8	High	Yes	Yes
gse	411.46	3.32	Moderately soluble	Yes	6	0	7	High	Yes	Yes

LogP, the oil-water partition coefficient of a molecule, is the logarithmic value of the ratio of the partition coefficients of a substance in the oily substance octanol and in water. A higher LogP value represents a higher lipophilicity of the compound, while the opposite situation represents a high hydrophilicity of the compound. 7 of the lead molecules were predicted to have moderate or good water solubility, but they still had a high LogP value (maintained around 3). This suggests that all lead molecules have a higher lipophilicity relative to water solubility.

Permeable glycoprotein (P-gp) is a drug pump protein located in the cell membrane that pumps foreign drugs out of the cell in an ATP-dependent manner and reduces the in vivo availability of the drug. All compounds, except i472b and i472e, are predicted to be substrates of P-gp.

Passive diffusion is the main mode of entry of small molecules into the brain, and therefore the blood-brain barrier permeability of a molecule is largely dependent on its lipophilicity. However, some of the P-gp substrate molecules are actively pumped out after entering the blood-brain barrier, which would greatly reduce the rate at which drug molecules enter the brain. Of the total molecules, i472a and gsc exhibited the best water solubility and low LogP index, but relatively, they were also predicted not to have blood-brain barrier permeability.

CYP family proteins are a series of vital hepatic enzymes that play a key role in the hepatic metabolism of drugs. Some of the drugs themselves are able to inhibit the activity of hepatic drug enzymes and thus prolong their own course of action in vivo. In the predicted results, all molecules were predicted to have CYP2D6 inhibitory properties. Combined with their good gastrointestinal absorption, it can be assumed that these lead molecules have excellent in vivo utilisation effects.

Lipinski’s five rules were most widely used in various virtual studies, and therefore the druggability of the full range of lead molecules was judged by Lipinski’s rules^25^. These rules include: molecular weight less than 500; number of hydrogen bond donors not exceeding 5; number of hydrogen bond acceptors not exceeding 10; number of rotatable bonds less than 10; and the logP of the oil-water partition coefficient (logP) of the compound not exceeding 5. The predictions showed that none of the molecules violated Lipinski’s five rules.

Finally, the potential toxicity of these candidate compounds was assessed using the Quantitative Structure-Toxicity Relationship (QSTR) model of the TOPKAT module and the results are shown in Table 8. In the predicted results, all compounds were predicted to be non-teratogenic. In the FDA-compliant mouse model, all of the i472 series compounds were predicted to be non-Rat carcinogenic, while the GS series compounds were predicted to be carcinogenic to male Rat. In addition, we predicted the cell line toxicity of the candidate compounds, and due to space constraints, this part of the results is shown in the Supplementary Material.

**Table 8.** Results of teratogenicity, carcinogenicity to male/female Rat tests for seven lead molecules.

Molecule	Ames_Prediction	Ames_Probability	Ames_TOPKAT Score	Carcinogen_Prediction (Male/Female Rat)	Rat_Male_Carcinogen_ Probability	Rat_Female_Carcinogen_ Probability
i472a	Non-Mutagen	0.68	−2.59	Non-Carcinogen/Non-Carcinogen	0.33	0.19
i472b	Non-Mutagen	0.63	−4.04	Non-Carcinogen/Non-Carcinogen	0.32	0.18
i472e	Non-Mutagen	0.69	−2.25	Non-Carcinogen/Non-Carcinogen	0.31	0.18
gsa	Non-Mutagen	0.60	−5.03	Single-Carcinogen/Non-Carcinogen	0.42	0.22
gsb	Non-Mutagen	0.53	−6.74	Single-Carcinogen/Non-Carcinogen	0.46	0.23
gsc	Non-Mutagen	0.65	−3.54	Single-Carcinogen/Non-Carcinogen	0.47	0.25
gse	Non-Mutagen	0.63	−4.14	Single-Carcinogen/Non-Carcinogen	0.37	0.25

### Molecular dynamics analysis

**Figure 6.**
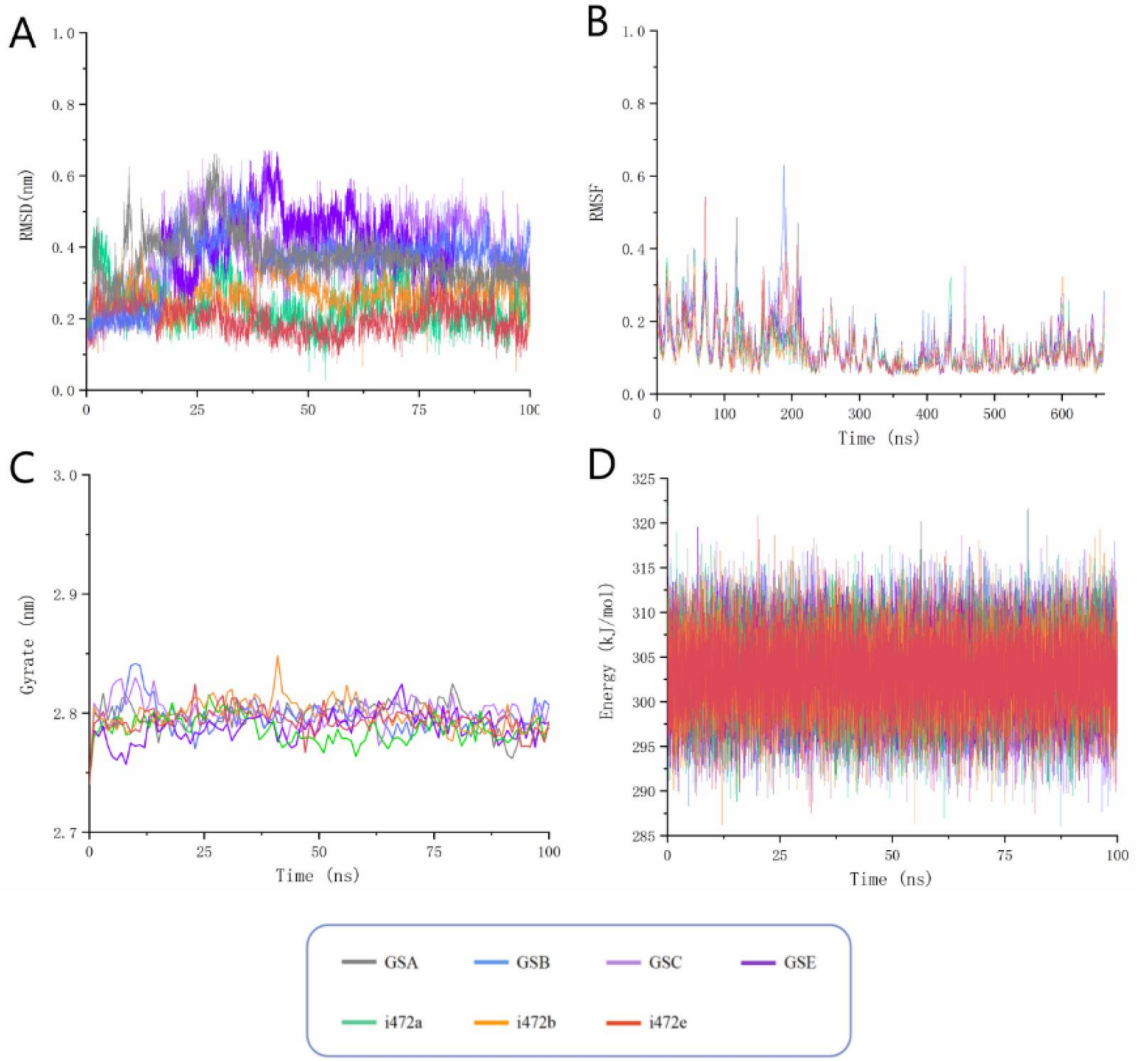
Molecular dynamics simulations of the seven candidate molecules in complex with ALOX15 protein. (A)fluctuations in the root mean square deviation of the ligand; (B) fluctuations in the root mean square fluctuation of the protein residues forming the complex; (C) fluctuations in the radius of gyration of the protein receptor; (D) fluctuations in the potential energy of the complex system.

As shown in Figure 6(A), the conformational fluctuations of the seven molecules remained relatively stable during the 100 ns molecular dynamics simulation, basically between 0.1–0.5 nm. Among them, the RMSD fluctuations of the three ligands, i472a/b/e, were always maintained at a relatively low level between 0.1–0.3 nm, and they also showed a similar fluctuation trend, which indicated that their structures were more stable in binding to the target. i472a/b/e, gsa/b/c/e, showed a lower fluctuation level within the first 20 ns, and although they increased in the following, their numerical levels were maintained at a more stable level, and in the second half of the simulation process, the RMSD values have been largely maintained between 0.35–0.4 nm. Overall, all the molecules have finally reached conformational stability.

Next, the conformational fluctuations of the receptor protein portion of the complex, i.e. the root-mean-square fluctuations (RMSF) of the residues, were analysed to determine the stability of the ligand-receptor binding. The results are shown in Figure 6(B). Firstly, the trends of the root mean square fluctuations of the seven ligand-receptor complexes overlapped well, which proved that our simulation method was reliable because the backbones of all the receptor proteins did not undergo any major changes or collapses. Second, the RMSF values of all proteins basically remained between 0.05 and 0.6, indicating that the receptor conformation remained stable throughout the process.

In addition, we calculated and evaluated the radius of gyration (Rg) of the receptor protein structure in the complex system as well as the total energy change of the complex system. The radius of gyration (Rg) indicates the stability and denseness of the protein structure. As shown in Figure 6(C), the average radius of gyration value of the proteins in the complex system stays around 2.79 nm, indicating that the structure of the proteins forming the complex remains dense throughout the simulation. The changes in the potential energy of the complexes are shown in Figure 6(D). The total potential energy of all the small-molecule ligand-protein receptor complexes remained stable throughout the dynamic simulation, and the estimated average total energy value was kept at 303 kJ/mol, which confirmed the stable binding of the novel ligands and proteins from the energy point of view.

**Figure 7.**
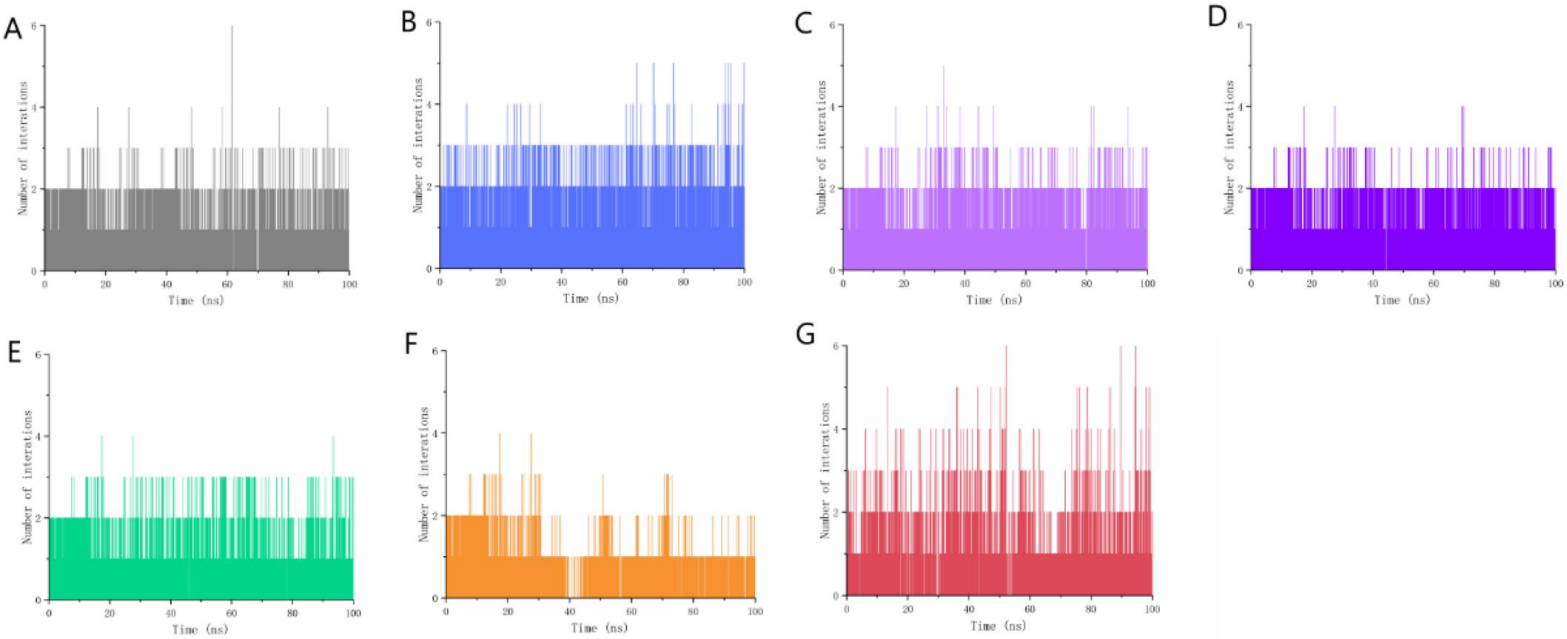
Number of interactions between 7 candidate molecules and ALOX15 protein over time. (A) Number of interactions between gsa and protein over time; (B) Number of interactions between gsb and protein over time; (C) Number of interactions between gsc and protein over time; (D) Number of interactions between gse and protein over time; (E) Number of interactions between ia and protein over time; (F) Number of interactions between ib and protein over time; (G) Number of interactions between i.e. and protein over time. number of interactions over time; (G) number of interactions between i.e. and proteins over time.

In order to verify that the ligand is consistently located within the protein active site throughout the simulation, we have also added hydrogen bonding analyses of the seven ligand-receptor complexes. We applied GROMACS program to monitor the number of interactions formed between the ligand and the protein throughout the simulation to determine whether the ligand was stably bound to the active pocket of the protein throughout. Since our candidate molecules did not form many hydrogen bonds with the protein and formed more Pi interactions, which are difficult to monitor, we considered an interaction to be formed when the distance between the atoms on the ligand and the protein was less than 0.35 nm at a given moment. As can be seen from Figure 7A–G, these candidate molecules remained relatively close to the protein for almost the entire 100 ns. This further verifies that these seven candidate molecules have stable interactions with the protein.

To verify the binding interactions between the seven candidate compounds and the ALOX15 protein, we conducted binding free energy calculations based on the Molecular Mechanics Poisson Boltzmann Surface Area Method (MM-PBSA) with a time step of 10 ns. The results, shown in Table 9, suggest that Ia/b/e have relatively stronger binding energies, which is consistent with our previous analysis of the dynamic trajectories. All seven compounds, however, have good target binding ability.

**Table 9.** Binding energy of between the candidate compounds and ALOX15.

Energy	Molecule	Features
Binding energy(kJ/mol)	gsa	−156.065 ± 42.274
	gsb	−162.429 ± 43.326
	gsc	−158.314 ± 39.992
	gse	−153.042 ± 40.982
	ia	−202.140 ± 37.789
	ib	−205.482 ± 38.842
	ie	−204.671 ± 41.347

## Discussion

In previous studies, ferroptosis has been recognised as an important pathway for non-programmed tumour cell death^26^. The ALOX family of proteins is essential for regulating the oxidative levels of cells, particularly ALOX12 and ALOX15. It is believed that these proteins induce peroxidation of the cytoplasmic membrane, resulting in oxidative damage, such as ferroptosis. Consequently, ALOX15 has become a primary target in the development of drugs to combat its anti-inflammatory and anti-ROS effects, which have been validated in cellular histological tests^27–29^. In the last ten years, the advancement of computer-aided drug design (CADD) technology and machine learning techniques have significantly improved drug discovery. This technology enables researchers to find lead compounds quickly and accurately without the need for extensive time and resources. Nonetheless, many CADD studies have been conducted on natural compounds, which have a great structural variety and potential active properties, but lack desirable drug-ready properties. This has caused researchers to expend too much effort in the early stages of drug development. Re-screening towards FDA-approved drug libraries for drug repurposing is a good response to this dilemma^30^.Recently, machine learning techniques have been used extensively in drug discovery. A virtual screening approach based on target structures, incorporating machine learning models derived from Bayesian and Recursive Partition algorithms, was utilised to select the three most promising target binding molecules from the FDA-validated compound structure database, namely OSI-930, SD-208 and GS-444217. OSI-930 is a potent inhibitor of the colony-stimulating factor 1 receptor (CSF-1R) which effectively inhibits the growth of HMC-1 cells. SD-208 is a TGF-βR inhibitor that boosts the anti-tumour response of myeloid and lymphocytes^31^. GS-444217 is a potent ATP-competitive apoptosis signal-regulated kinase 1 (ASK1) inhibitor that inhibits stress-induced apoptosis^32^^,^^33^. Taken together, the three compounds have been used to treat diseases related to tumours and immunity, but do not share a common target. In the study, it was initially assumed that they may have good binding activity to ALOX15, similar to the known inhibitor i472. After further structural improvement, additional target inhibitors with novel structures were found. ADMET characterisation and molecular dynamics calculations with physiological concentrations in salt solution showed that these molecules have potential for in vivo application. Of them, gsc has good ADMET properties and kinetic binding stability, which may make it advantageous for further development of ALOX15 inhibitors. However, virtual screening can only predict the physical-chemical binding ability of the molecule, but not its intrinsic activity towards the target protein, which needs to be tested in further *in vitro* experiments.

## Material and methods

### Virtual screening

6140 molecules from the FDA Medicinal Compound Database were used for the preliminary virtual screening, using Alphafold’s human ALOX15 model (P16050-LOX15_HUMAN) as the receptor structure, and the active site was defined referring to the literature published by Meng et al^34^. on critical residues in the ALOX15 binding pocket. The high-throughput screening based on fast docking was performed using the Libdock module of the Discovery Studio platform version 2019, with 100 Hotspots randomly generated in the defined active docking space. Up to 100 spatial conformations were generated for each compound by rapid conformation methods, and these conformations were coincided to spatial hotspots, which were finally determined by the number and type of ligand-receptor interactions. Each successful docked conformation was scored according to the number and type of ligand-receptor interactions.

### Machine learning - QSAR

#### Data set preparation, characterisation and down-scaling

For the section on data set characterisation and dimensionality reduction see the Supplementary Material.

#### QSAR model training and validation

Naive Bayesian and decision tree models are the two most widely used machine learning classifier models. The Naive Bayesian classifier is an efficient machine learning algorithm based on Bayes’ theorem, which is commonly used to classify training sets with a large number of samples due to its immunity to random noise during training. Considering that the ALOX15 inhibitor database we chose contains 581 compounds, and that the structures of these compounds vary widely according to the results of the chemical space characterisation of the data set, the use of the Naive Bayesian model is more capable of shielding the influence of the noisy points in the database to obtain more accurate classification results compared to other models^35^; Recursive Partitioning (RP) is a commonly used multivariate analysis method to classify samples within a data set by constructing one or more decision trees. A single decision tree contains different numbers of nodes within it depending on the depth of the tree set, which consists of input significant variables, and the RP model classifies examples into different categories based on whether an example within the data set meets the characteristic conditions of that node. In past practice, RP forest models have often demonstrated better classification performance than support vector machines (SVMs) and artificial neural networks (ANNs)^36^, and are less prone to over-fitting than single tree models. Considering that a single machine learning model has its drawbacks, we used both models in this study to get higher screening accuracy. For all these reasons, we chose the RP forest model as a complement to the NB model.

#### Naive Bayesian model

Using the QSAR modelling module of the Discovery Studio platform, binary values of biological activity (active as "1" and inactive as "0") defined in the previous data set preparation step were used as input dependent properties. The Bayesian theorem is given by the following equation (Equation (1)):

(1) $$P(A|B)=\frac{P(B|A)P(A)}{P(B)}$$

Where A represents the model’s classification of the data and P(A) is the prior probability that the classification predicts the data; B represents the actual value of the data and P(B) is the prior probability of the value. p(B|A) is the posterior probability that classification B is predicted to be true when the assumption that classification A holds, and P(A|B) is the posterior probability that classification A is predicted to be true given the observed data B. We constructed Naive Bayesian models using the Create QSAR Model function of Discovery studio. The 12 highest weighted descriptors obtained from the principal component analysis were used as input independence properties, while the structures of the training set compounds were characterised with ECFP_6 fingerprints. During the construction of Naive Bayesian model, we applied the Laplace correction method to eliminate, as far as possible, the over-fitting error caused by the accidental separate appearance of specific molecular features in the active/inactive data set.

The quality of the model is assessed by calculating mertics such as Sensitivity, Specificity, Precision, F-Measure and MCC values, which are derived from a secondary calculation of the true/false positive and negative rates of the model classification results. The calculation formulae are shown below:

(2) $$\text{Sensitivity}=\text{Recall}=\frac{\text{TP}}{\left(\text{TP}+\text{FN}\right)}$$

(3) $$\text{Specificity}=\frac{\text{TN}}{\left(\text{FP}+\text{TN}\right)}$$

(4) $$\text{Precision}=\frac{\text{TP}}{\left(\text{TP}+\text{FP}\right)}$$

(5) $$F-\text{Measure}=\frac{(1+\alpha 2)*\text{precision}*\text{recall}}{\text{precision}+\text{recall}}$$

(6) $$\text{MCC}=\frac{\text{TP}*\text{TN}-\text{FP}*\text{FN}}{\surd (\text{TP}+\text{FP})(\text{TP}+\text{FN})(\text{TN}+\text{FP})(\text{TN}+\text{FN})}$$

Sensitivity represents the percentage of examples that were correctly classified as positive by the classifier model for each example in the data set (Equation (2)), namely the true-positive rate. Specificity represents the percentage of negative examples that were correctly classified (Equation (3)), while Precision represents the percentage of true positive examples out of all instances of true positives and those that were falsely predicted as positive (Equation (4)).

F-Measure is the weighted average of sensitivity and specificity (Equation (5)). Different α value give different weights to sensitivity and specificity, and in the evaluation of machine learning models, the α value is often set to 1 (i.e. the same weighting factor is given to both) and it is referred to as the F-1 score. In previous practice, F-1 score has been positively correlated with the reliability of the model.

Matthews correlation coefficient (MCC) is a kind of Pearson correlation coefficient (Equation (6)) that are calculated based on a weighting matrix and ranges from [−1, 1], where a higher positive value indicates better classification performance and correspondingly a lower negative value indicates a greater deviation of the predicted result from the actual classification.

#### Recursive partition classifier

Considering that a single decision tree is more prone to over-fitting, we constructed a forest model consisting of 10 trees. The construct of the forest model was based on the recursive partition algorithm in order to compare its performance with that of the Bayesian model. The RP model was developed in the Discovery Studio platform, and the 12 molecular property descriptors calculated in the PCA step and the ECFP_6 fingerprint were used as the inputs to build the model. The minimum number of samples per node was set to 10, and the nodes were partitioned using the Gini algorithm. The maximum tree depth was set to 20. And the internal performance was tested by the bagging method.

For all models, external validation of the models was performed using a pre-segmented test set. Finally, the combined best NB and RP models were subjected to a secondary screening of the dummy screened candidate molecules to further narrow the range of lead compounds considered.

### Refined docking analysis

The CDOCKER module^37^ of the Discovery Studio was used to perform further refined docking analysis for candidate compounds. The binding energies between ligands and receptors were measured using the CDOCKER ENERGY and CDOCKER INTERATION ENERGY metrics, as well as the type and amount of compound-protein interactions, to evaluate the binding situation of compounds to receptors. A 1000 steps dynamic simulation was conducted on the compounds in the CHARMM force field to generate their conformations, with a target temperature of 1000 K and pose cluster radius set to 0.1. 7000 steps simulation was executed on the system during the docking process. The simulation was run on the system during the whole docking process, where the temperature was first raised to 700K by a 2000 steps process and then lowered to 300K by another 500 steps process.

### Fragment replacement of the lead compounds

Based on the results of refined docking, we optimised the fragment substitution for each of the three candidate compounds and the positive control compound i472. The binding conformation of the compound structure within the target was used as the bases for the fragment replacement for lead compound structures that are not interacting with target residues. The compounds were first energetically minimised using the CHARMM force field, followed by fragment replacement calculations performed using Discovery Studio’s default fragment library (1,495,478 fragments in total). The number of rings/aromatic rings as well as the surface area of molecules were used as the properties for fragment similarity calculation, and the fragments with the highest physical and chemical similarity to the original fragments and the best overall novelty were selected to optimise molecular structures for substitution. The optimised molecules are Pareto-ranked by the number of interactions with proteins, Lipinski violations, receptor collisions and fragment novelty (number of chain combinations, number of double and aromatic bonds and number of N, S and O atoms). Finally, the molecules were re-docked to the target with the Libdock module and the best novel molecule was selected based on docking score and number of favourable interactions.

### ADMET property studies

The absorption, partitioning, metabolism, excretion and toxicity (ADMET) properties of compounds often determine their value for in vivo use. In general, ADMET assays are commonly carried out in animal models, but can be time-consuming and costly. In recent years, an increasing number of online compound property prediction tools have been developed to deal with this situation. At this stage, an online tool SwissADME^38^ (http://www.swissadme.ch/) was applied to assess the properties of screened and modified candidate molecules to predict their drug-llikeness characteristics, including lipid-water distribution coefficient, blood-brain barrier permeability, intestinal absorption, P-gp inhibition, hepatic drugase inhibition and drug-like properties. The TOPKAT module of the Discovery Studio^39^ was used as a predictor for the teratogenicity and carcinogenicity of the compounds.

### Molecular dynamics analysis

Molecular docking methods only investigate on the theoretical binding ability between compounds and receptors under ideal stand-alone conditions, and thus good docking results do not fully describe the target binding ability of lead compounds in realistic situations. Molecular dynamics simulations (MD) are often used to monitor the stability of protein-ligand binding systems under certain temperatures, pressures and salt solution environments in simulated organisms. Therefore, we have calculated the conformational fluctuations of the complexes formed by the binding of seven lead molecules to the target over a 100 ns time span. The dynamic target binding potential of all candidate compounds was analysed in terms of the conformational fluctuations and surface solvent area of the ligands, the protein radius of gyration, and the binding potential, respectively. Firstly, PDB files of the seven leads and receptor proteins were constructed and exported from the Discovery Studio platform. GAFF force field-based ligand topologies were constructed using the ACPYPE online server^40^ (https://www.bio2byte.be/acpype/). Version 2019 of the GROMACS program^41^ was used to construct topology files of proteins and MD calculations, applying the AMBER99SB-ILDN force field and TIP3 water model was used to construct topology files for the proteins. A cubic box with a radius of 1.2 nm was constructed to accommodate topological systems of protein-receptor complexes, and the box was filled with SPC216 water models to simulate aquatic environments. Corresponding amounts of sodium and chloride ions were also added to the solvent system to balance charges. After the simulation system was successfully constructed, a 50,000 steps energy minimisation calculation was operated at a simulated temperature of 300K. Subsequently, the receptor, ligand and solvent in the system chamber were equilibrated under constant temperature and volume (NVT) and constant temperature and pressure (NPT) conditions with a duration of 25 ps and a step size of 25,000 steps. Finally, MD simulations of 100 ns duration were ran for the system. Root mean square deviation (RMSD) and root mean square fluctuation (RMSF) of atomic positions of the systems were analysed. In addition, the radius of gyration (Rg), total potential energy variation curves, and number of hydrogen bonds were collected for each system.

Molecular Mechanics/Poisson Boltzmann Surface Area (MM/PBSA) method is widely used to calculate the receptor-ligand binding free energy ^42^. The basic principle is to calculate the difference between the binding and unbinding free energies comparing different solvated conformations of the same molecule (as shown in Equation (7), G_Complex_ represents the free energy of the protein-ligand complex, G_protein_ represents the free energy of the protein in the solvent, and G_ligand_ represents the free energy of the ligand in the solvent). We extracted one frame of conformation from the previously obtained kinetic trajectories at the same time difference intervals, and finally extracted a total of 10 ns time-length trajectories for binding free energy calculation.

(7) $$G_{\text{binding}}=G_{\text{protein}}-(G_{\text{complex}}+G_{\text{ligand}})$$

## Conclusion

In this study, a combination of structure-based and machine learning virtual screening was employed to identify three FDA-approved drugs as potential lead compounds for targeting ALOX15. Subsequently, structure substitution optimisation based on docking results and fragment usage was used to generate compounds with improved target-binding capability. Moreover, ADMET property predictions and molecular dynamics simulations suggest that these lead molecules could have promising in vivo applications. In conclusion, the newly identified lead compounds offer potential for the development of ALOX15 inhibitors for treating inflammation-mediated injury and degenerative diseases associated with ferroptosis.

## Supplementary Material

Supplemental Material

## Data Availability

The data that support this study are available from the corresponding author upon reasonable.
