# Supplementary material for: Development of novel ALOX15 inhibitors combining dual machine learning filtering and fragment substitution optimisation approaches, molecular docking and dynamic simulation methods
Source: J Enzyme Inhib Med Chem. 2024 Jan 12;39(1):2301756. doi: 10.1080/14756366.2024.2301756 (PMC10791093; doi:10.1080/14756366.2024.2301756)
Supplement: Supplemental Material [file IENZ_A_2301756_SM4850.pdf]

Supplementary Material

Reliability Verification of Alpha Fold ALOX15 Structure

Structure and sequence superposition

To illustrate the reliability of the Alpha fold ALOX15 structure, we aligned the Alpha fold's Homo sapiens ALOX15 protein model with the rabbit-derived ALOX15 PDB structures 1LOX and 2P0M, respectively, and compared the differences between their residue sequences and the Root Mean Square Deviation (RMSD) between their α-C chain backbones. As shown in Supplementary Table 1 and Supplementary Figure 1A-B, the results indicate that the spatial mean square deviation of the ALOX15 protein provided by the Alpha fold stacked with the two rabbit ALOX15 structures is very small, and all of them are less than 1 Å. In the supplementary figures 1C-D, it can be seen that the sequence similarity between the ALOX15 structure provided by Alpha fold and the two PDB structures are both very high (in the upper and lower 90%). These analyses indicate that the structures provided by Alpha fold are highly credible.

Supplementary Table 1. Comparison of spatial differences between PDB rabbit-derived ALOX15 structures and Alpha fold human-derived ALOX15 structures.

PDB structure	RMSD between Alpha fold structure（angstrom）	Sequence similarity
1LOX	0.74 Å	89.40%
2P0M	0.81 Å	91.60%

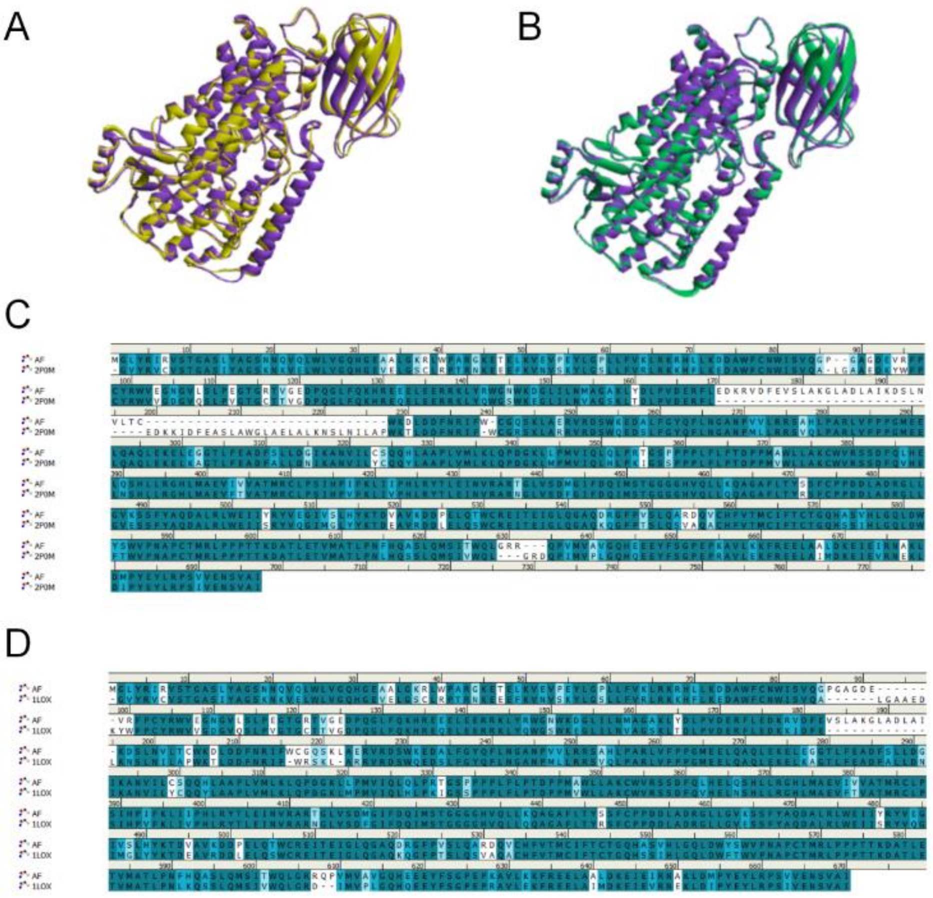

Supplementary Figure 1. Alpha fold Homo sapiens ALOX15 protein model aligned with the rabbit-derived ALOX15 PDB structures 1LOX and 2P0M. (A)Alpha fold ALOX15 3D structure aligned with PDB structure 2P0M; (B)Alpha fold ALOX15 3D structure aligned with PDB structure 1LOX; (C)Alpha fold ALOX15 structural sequence versus PDB 2P0M structural sequence; (D)Alpha fold ALOX15 structural sequence versus PDB 1LOX structural sequence.

Docking and dynamic Validation of the Alpha Fold ALOX15 Structure with Inhibitors

We used an additional 4 well-known ALOX15 inhibitors to dock with the ALOX15 structure provided by Alpha fold to validate our docking scheme. Docking energy scores and number of interactions are shown in Supplementary Table 2. In addition, we performed dynamic simulations of the docked complexes. As can be seen in Supplementary Figure 2, the average value of the RMSD for the four selected compounds was maintained between 0.2-0.4 nm over the 100 ns time duration and reached equilibrium during the latter part of the dynamic simulation. For the protein residues, they were maintained at a lower value (average value around 0.18 nm) throughout the duration of the time length. Taken together, the results of the all of these

inhibitors were predicted to interact more favorably with the Alpha fold structure, implying that our choice of alpha fold structure is reliable.

Supplementary Table 2. Docking results of four ALOX15 inhibitors with Alpha fold ALOX15 structures

ALOX15 inhibitors	Libdock score	CDOCKER ENERGY	CDOCKER INTERACTION ENERGY	Number of favourable interactions
RS7	95.9436	-18.562	-29.8922	5
ML351	87.7259	-13.0257	-21.5808	13
Phenidone	81.1910	-11.1346	-24.2905	6
Zileuton	98.5783	-25.1044	-28.7782	9

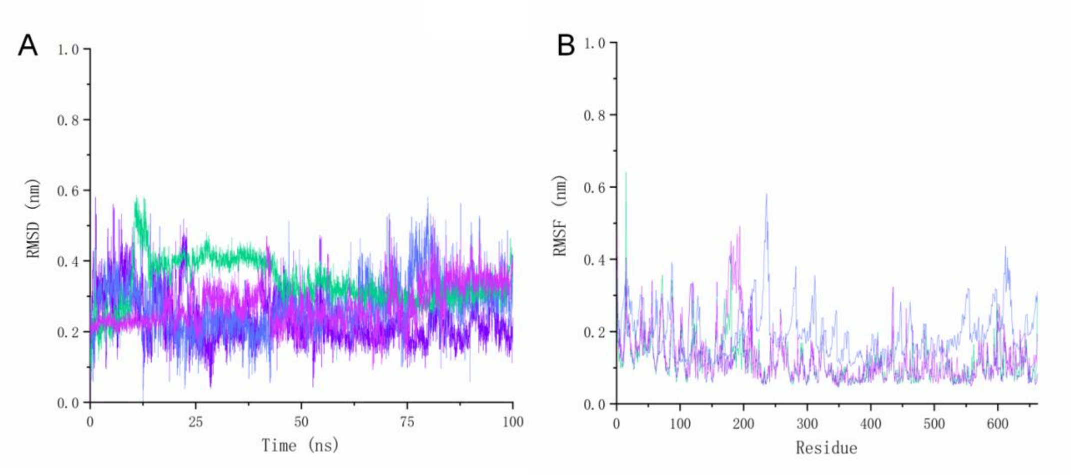

Supplementary Figure 2. Dynamic simulation results of docking complexes of the Alpha Fold ALOX15 structure with four selected ALOX15 inhibitors. (A) Dynamic RMSD results of the Alpha Fold structure in complex with selected inhibitors; (B) Dynamic RMSF results of the Alpha Fold structure in complex with selected inhibitors.

Characterization of the drug-like properties of the database

For the FDA database drugs used for screening, we provide initial characterization of their Lipinski-like drug properties in Supplementary Table 3: 799 molecules had more than 10 hydrogen bond donors, 693 molecules had more than 5 hydrogen bond acceptors, 1,192 molecules had an AlogP greater than 5; and 501 small molecules had a molecular weight of greater than 500.After the initial Libdock screening, the proportion of poorly drug-like molecules in the candidate After the initial Libdock screening, the proportion of molecules with poor drug similarity in the compound database dropped dramatically, which greatly reduced our cost of subsequently excluding non-drug molecules.

Supplementary Table 3. Distribution of drug-like properties of FDA drug libraries before/after Libdock screening.

Lipinski properties/Number of violated molecules	FDA database molecules	Molecules filtered by libdock
Hydrogen bond donor (＞10)	799	5
Hydrogen bond acceptor (＞5)	693	23
Molecular weight (＞500)	501	7
AlogP (＞5)	1192	67

Data set preparation and characterization

In order to determine whether the pre-divided training set and test set molecules have an averaging chemical space distribution, FCFP/ECFP/FPFP/EPFP_6 fingerprints were calculated for each training set/test set molecule separately and their overlapping degree was observed. During the preparation and characterization of the data set, in addition to the use of Tanimoto-based molecular fingerprints, we additionally used a number of common descriptors of molecular physical/chemical properties to construct the assigned dimensions and further create the attribute space. These descriptors are ALogP, Molecular Weight, Number of H-Donors, Number of H-Acceptors, Number of Rotatable Bonds, Number of Aromatic Rings, Number of Rings, Number of Number of Fragments and Molecular polar surface area.Subsequently, each molecule uses a descriptor as a positional coordinate and the distribution is mapped in this multidimensional property space. Thus, molecules located at different spatial coordinates have their specific corresponding properties. The results are shown in Supplementary Figure 3. In the chemical spatial distribution descriptions based on four fingerprints, a uniform scattering state was reached for both training and test set

molecules, indicating that no significant physical-chemical property skewing occurred during the random partitioning of the data set.

The diversity metrics of all 581 molecules were further calculated to express the specificity of each molecule in the data set separately in terms of molecule assemblies, fingerprint distance and property distance. The results are shown in Supplementary Table 4. Number of Assemblies is defined as the total number of assemblies divided by the number of molecules. It is a macroscopic measure of the number of similarly structured clusters formed by all molecules within the data set. The average number of molecular clusters within our data set is only 0.34, indicating that only a very small number of molecules form structurally similar clusters. We calculate the distance between fingerprints based on the Tanimoto coefficient, where the value of the distance is defined as "1-similarity". In general, smaller distances represent greater structural similarity. On the contrary, if the average molecular fingerprints we calculated have a large distance (0.88), then the structural similarity between them is low. Property distance is defined as the Euclidean distance of the specified numerical properties for every pair of molecules. It can be seen that the average property distance is in a low value of 1.28, indicating that the chemical spatial distribution between these molecules is relatively sparse and suitable for training and testing of machine learning models.

Supplementary Table 4. Diversity metrics for the ALOX15 inhibitor data set.

Diversity Metrics	Value
Number of Assemblies	0.34
Fingerprint Distance （average）	0.88
Property Distance （average）	1.29

*The data in the table are averages of the indicators for molecules in the data set.

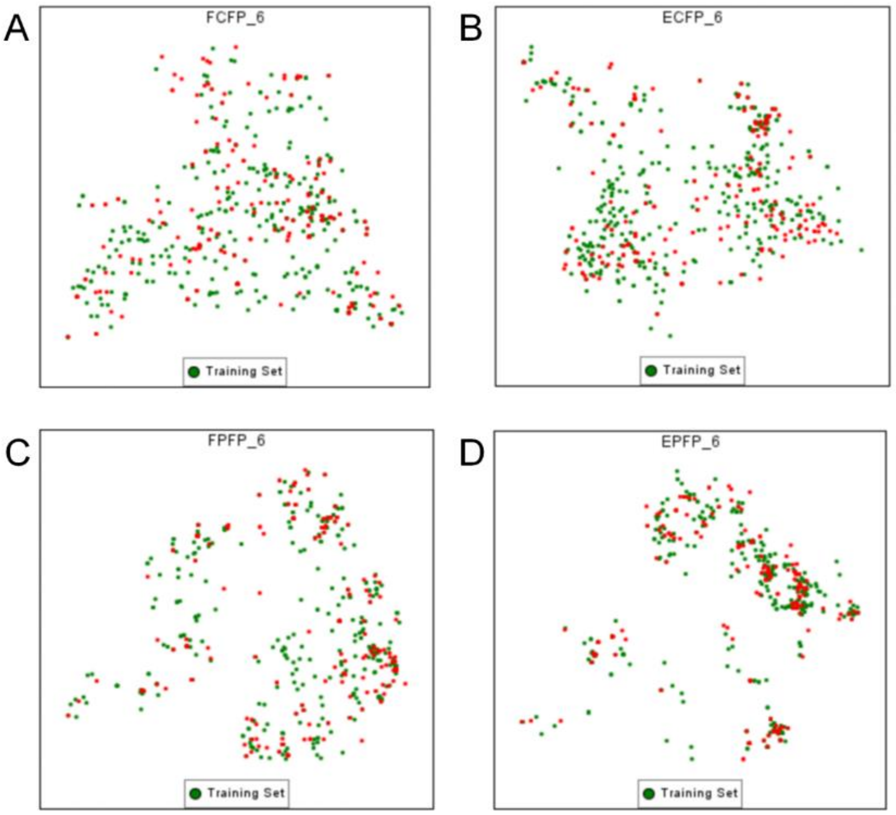

Supplementary Figure 3. Spatial distribution of training set-test set molecule chemistry used to build the machine learning QSAR model. The training set molecules are shown in green, while the test set molecules are in red. (A) Chemical space distribution calculated based on FCFP_6 (Functional classed Extended Connectivity Fingerprint_6); (B) Chemical space distribution calculated based on ECFP_6 (Atom type Extended Connectivity Fingerprint_6); (C) Chemical space distribution calculated based on FPFP_6 (Functional classed Extended Connectivity Fingerprint_6). (D) Chemical spatial distribution calculated based on EPFP_6 (Atom type Path-Based Fingerprint_6).

Data down-scaling: principal component analysis

Fifty-five commonly used descriptors of physicochemical properties were calculated for the entire data set, and 12 key molecular descriptors were derived by principal component analysis (PCA): ALogP, Molecular_Weight, Num_H_Donors, Num_H_Acceptors, Num_Rotatable Bonds, Num_Aromatic Rings, Num_Rings, Num_Atoms, Num_Fragments, Molecular_Surface Area, Molecular_Polar Surface Area, and Molecular_Fractional Polar Surface Area. Different weights were assigned

to these descriptors and five principal components (PCs) were obtained with the constituent descriptors and coefficient correlation equations , composition and scale factors, variance explained for each descriptor are presented in Supplementary Table 5. It can be seen that the number of molecular fragments has a greater weight in the composition of PCs compared to the other descriptors, indicating that the number of fragments that make up each molecule correlates with compound properties. The variance explained values represent the weight of each PC, with larger values indicating that the corresponding PC is of greater importance. The spatial distribution of the chemistry of PC1-PC3 is shown in Supplementary Figure 4.

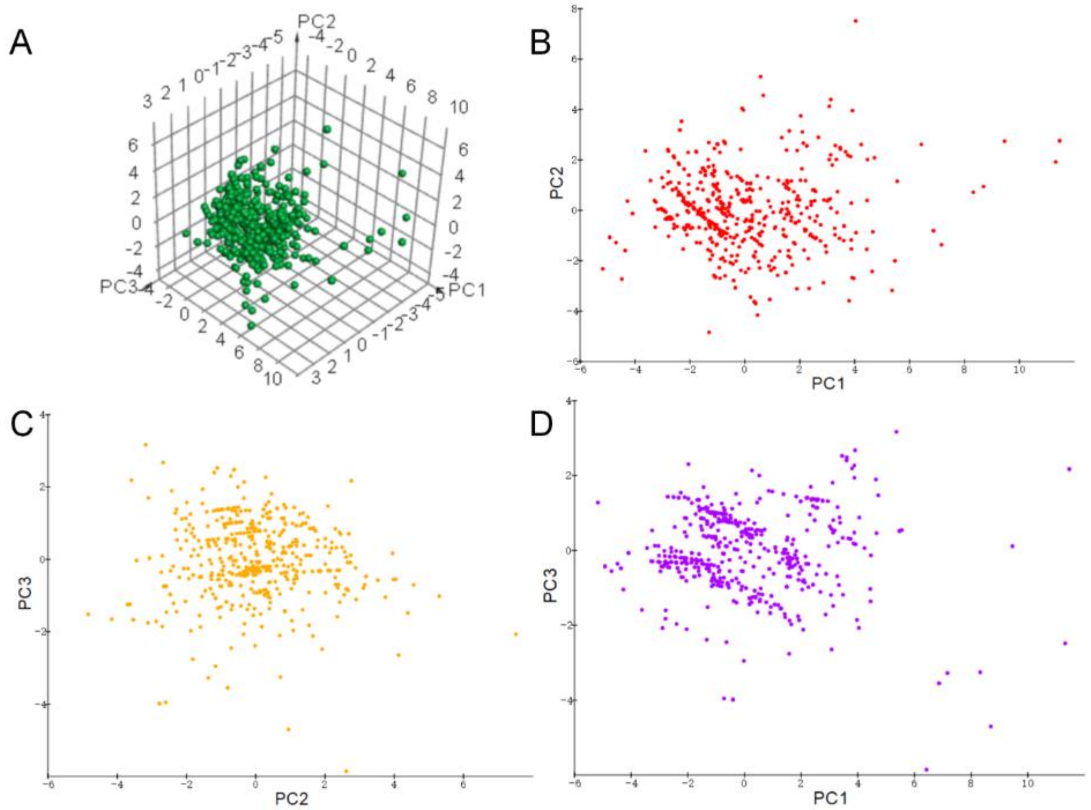

Supplementary Figure 4. Chemical space distribution of the three principal components PC1-PC3 obtained by dimensionality reduction. (A) Scatter plot of PC1-PC2-PC3 in 3D right-angle coordinates; (B) Chemical space plane distribution of PC2-PC1; (C) Chemical space plane distribution of PC3-PC2; (D) Chemical space plane distribution of PC3-PC1.

Supplementary Table 5. Five principal component equations obtained by degrading the ALOX15 inhibitor data set.

Principle Component	Equation	Variance Explained	Total Variance Explained
PC1	PC1 = -8.8466 + 0.16829 * [ALogP] + 0.0033936 * [Molecular_Weight] + 0.14535 * [Num_H_Donors] + 0.21739 * [Num_H_Acceptors] + 0.095361 * [Num_RotatableBonds] + 0.27641 * [Num_Rings] + 0.23753 * [Num_AromaticRings] - 0.45118 * [Molecular_FractionalPolarSurfaceArea] + 0.060307 * [Num_Atoms] + 0.003812 * [Molecular_SurfaceArea] + 0.010858 * [Molecular_PolarSurfaceArea] + 0.94693 * [Num_Fragments]	0.462	0.462
PC2	PC2 = -4.4141 - 0.22269 * [ALogP] - 1.2558e-003 * [Molecular_Weight] + 0.28091 * [Num_H_Donors] + 0.1984 * [Num_H_Acceptors] - 6.3259e-002 * [Num_RotatableBonds] + 0.08836 * [Num_Rings] + 0.12841 * [Num_AromaticRings] + 8.2406 * [Molecular_FractionalPolarSurfaceArea] - 1.0058e-002 * [Num_Atoms] - 1.4042e-003 * [Molecular_SurfaceArea] + 0.014532 * [Molecular_PolarSurfaceArea] + 2.0577 * [Num_Fragments]	0.206	0.668
PC3	PC3 = 2.2138 - 1.826e-002 * [ALogP] - 3.2044e-004 * [Molecular_Weight] - 0.24264 * [Num_H_Donors] - 5.3332e-002 * [Num_H_Acceptors] - 0.11976 * [Num_RotatableBonds] + 0.63759 * [Num_Rings] + 0.55467 * [Num_AromaticRings] - 0.69959 * [Molecular_FractionalPolarSurfaceArea] + 0.0027919 * [Num_Atoms] - 1.343e-003 * [Molecular_SurfaceArea] - 5.6858e-003 * [Molecular_PolarSurfaceArea] - 3.0631 * [Num_Fragments]	0.108	0.776
PC4	PC4 = -14.972 - 3.6088e-002 * [ALogP] - 1.4454e-004 * [Molecular_Weight] - 0.34073 *	0.086	0.862

	[Num_H_Donors] - 4.9591e-003 * [Num_H_Acceptors] - 4.2917e-003 * [Num_RotatableBonds] + 0.13243 * [Num_Rings] - 4.9768e-002 * [Num_AromaticRings] - 0.42177 * [Molecular_FractionalPolarSurfaceArea] + 0.0015219 * [Num_Atoms] + 0.00027824 * [Molecular_SurfaceArea] + 0.0011494 * [Molecular_PolarSurfaceArea] + 15.42 * [Num_Fragments]		
PC5	PC5 = -4.4448 + 0.12326 * [ALogP] + 0.0021718 * [Molecular_Weight] + 0.53628 * [Num_H_Donors] - 0.11408 * [Num_H_Acceptors] - 0.19111 * [Num_RotatableBonds] + 0.080558 * [Num_Rings] - 0.1015 * [Num_AromaticRings] - 2.7019 * [Molecular_FractionalPolarSurfaceArea] - 4.5398e-003 * [Num_Atoms] - 1.8506e-004 * [Molecular_SurfaceArea] - 4.8766e-003 * [Molecular_PolarSurfaceArea] + 4.5686 * [Num_Fragments]	0.053	0.915

Supplementary Table 6. Molecular descriptors computed for machine learning model building.

Calculated Properties
ALogP,LogD,Molecular_Mass,Molecular_Solubility,Molecular_Weight,QED,QED_ALERTS,QED_ALOGP,QED_AROM,QED_HBA,QED_HBD,QED_MW,QED_PSA,QED_ROTB,SAs

core,SAscore_Complexity,SAscore_Fragments,VSA_TotalArea,HBA_Count,HBD_Count,Num_AromaticBonds,Num_AromaticRings,Num_AtomClasses,Num_Atoms,Num_Bonds,Num_ChainAssemblies,Num_Chains,Num_ComplexedFragments,Num_ExplicitAtoms,Num_ExplicitBonds,Num_Fragments,Num_H_Acceptors,Num_H_Acceptors_Lipinski,Num_H_Donors,Num_H_Donors_Lipinski,Num_Hydrogens,Num_Rings,Num_RotatableBonds,Molecular_FractionalPolarSASA,Molecular_FractionalPolarSurfaceArea,Molecular_PolarSASA,Molecular_PolarSurfaceArea,Molecular_SASA,Molecular_SAVol,Molecular_SurfaceArea, ES_Count_aaCH, ES_Count_aaN, ES_Count_aaO,ES_Count_aaNH, ES_Count_aaS, ES_Count_aaaC, ES_Count_aasN, ES_Count_aasC, ES_Count_dCH2, ES_Count_dNH, IsChiral,

Machine Learning validation

Supplementary Table 7. False positive rate of Naive Bayesian and Recursive Partition models in different validation methods.

Validation/Model	Naive Bayesian	Recursive Partition
10-fold validation	0.23	--
Test set validation	0.10	0.20
In-bag validation	--	0.17
Out-of-bag validation	--	0.33
Average False positive rate	0.17	0.23

Supplementary Table 8. Validation results of machine learning re-screening of seven candidate molecules

Candidate molecule	Recursive Partition score	Naive Bayesian score
gsa	0.55	0.78
gsb	0.53	0.81
gsc	0.56	0.75
gse	0.55	0.76
ia	0.96	0.86
ib	0.93	0.81
ie	0.92	0.83

*If a molecule has a score greater than 0.5 in the screen, it is classified as active.

Refine docking

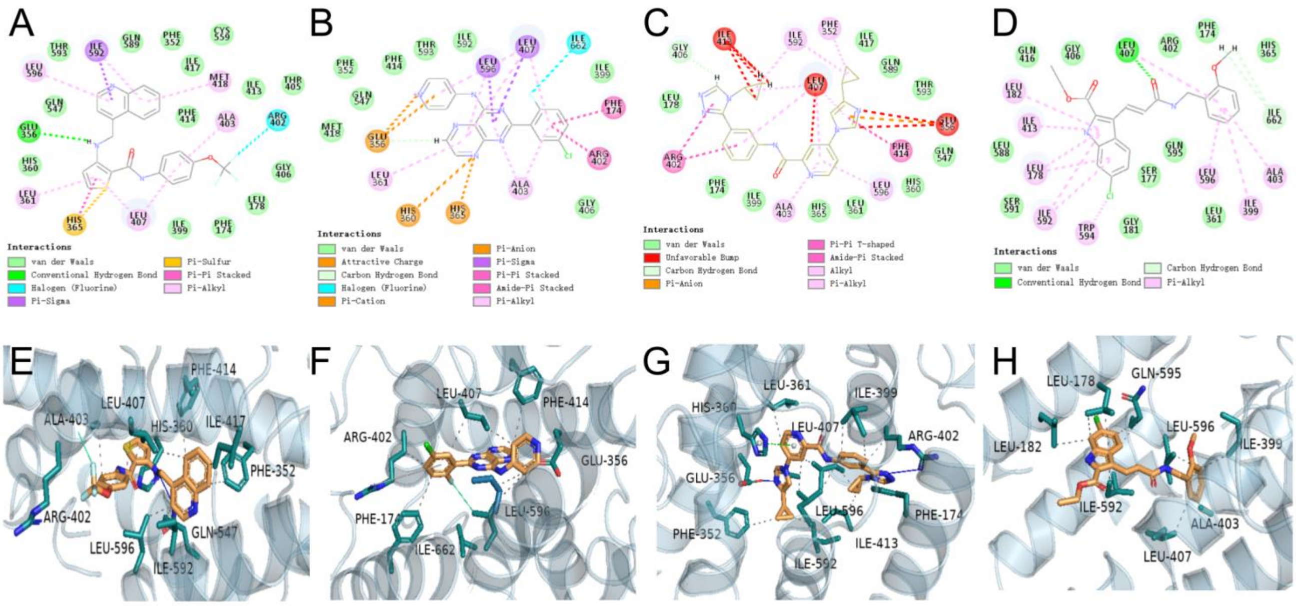

Supplementary Figure 5. Schematic diagram of the interaction of the three lead molecules and the positive control i472 with ALOX15 obtained from the screening. (A)Plan view of the interaction of OSI-930 with ALOX15; (B) Plan view of the interaction of SD-208 with ALOX15; (C) Plan view of the interaction of GS-444217 with ALOX15; (D) Plan view of the interaction of i472 with ALOX15; (E) 3D interaction of OSI-930 with ALOX15; (F) 3D interaction of SD-208 with ALOX15; (G) 3D interaction of GS-444217 with ALOX15; (H) 3D interaction diagram of i472 with ALOX15.

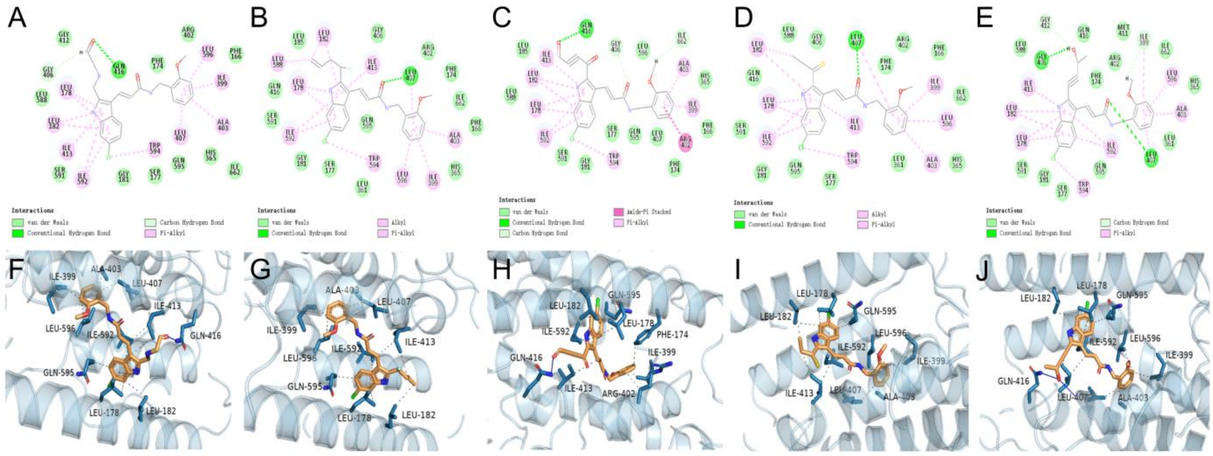

Supplementary Figure 6. Schematic diagram of interactions between i472a-e and ALOX15.（A）

Plane diagram of the interaction of i472a with ALOX15; (B) Plane diagram of the interaction of i472b with ALOX15;(C) Plan view of the interaction of i472c with ALOX15; (D) Plan view of the interaction of i472d with ALOX15; (E) Plan view of the interaction of i472e with ALOX15; (F) 3D interaction of i472e with ALOX15; (G) 3D interaction of i472e with ALOX15; (H) 3D interaction of i472e with ALOX15; (I) 3D interaction diagram of i472e with ALOX15; (J) 3D interaction diagram of i472e with ALOX15.

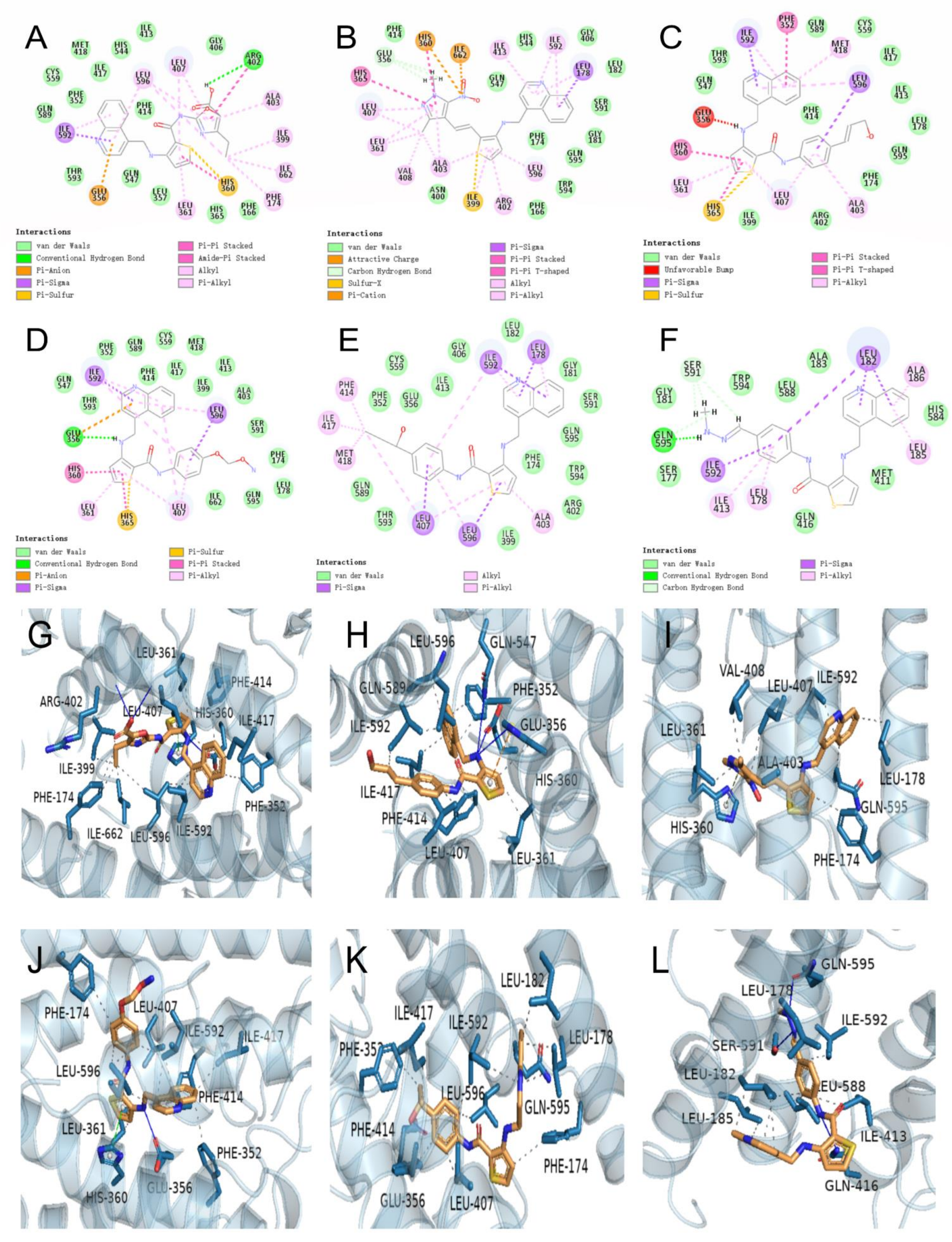

Supplementary Figure 7. Schematic diagram of the interaction between osia - osif and the target. (A) Plane of interaction between osia and ALOX15; (B) Plane of interaction between osib and

ALOX15;(C) Plane diagram of the interaction between osic and ALOX15; (D) Plane diagram of the interaction between osid and ALOX15; (E) Plane diagram of the interaction between osie and ALOX15; (F) Plane diagram of the interaction between osif and ALOX15; (G) 3D diagram of the interaction between osia and ALOX15; (H) 3D diagram of the interaction between osib and ALOX15; (I) 3D diagram of the interaction between osic and ALOX15; (J) 3D diagram of the interaction between osid and ALOX15; (K) 3D diagram of the interaction between osie and ALOX15; (L) 3D diagram of the interaction between osif and ALOX15.

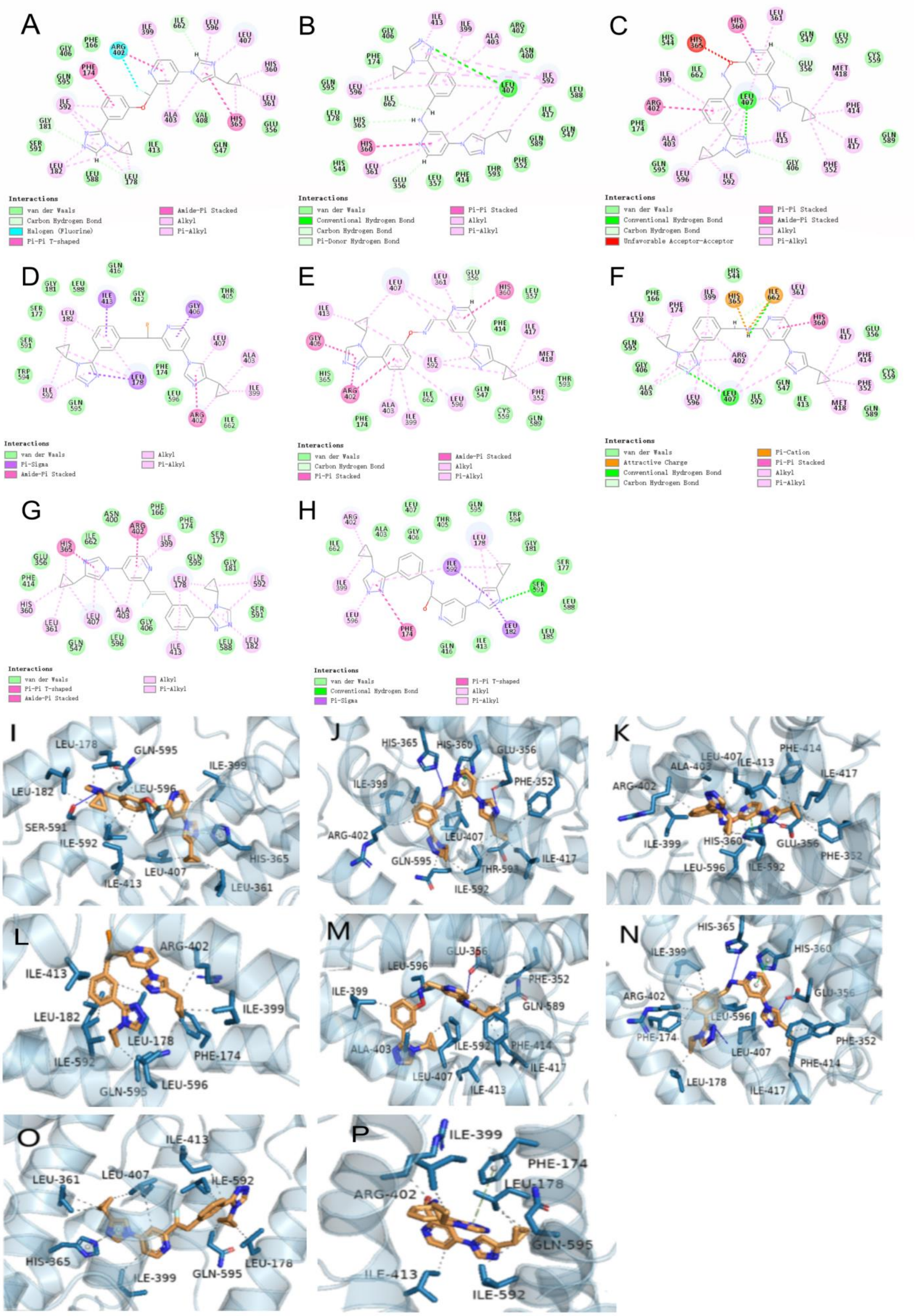

Supplementary Figure 8. Schematic diagram of the interaction between gsa - gsh and the target. (A)

Plane of interaction between gsa and ALOX15; (B) Plane of interaction between gsb and ALOX15; (C) Plane diagram of the interaction between gsc and ALOX15; (D) Plane diagram of the interaction between gsd and ALOX15; (E) Plane diagram of the interaction between gse and ALOX15; (F) Plane diagram of the interaction between gsf and ALOX15; (G) Plane diagram of the interaction between gsg and ALOX15; (H) Plane diagram of the interaction between gsh and ALOX15 planes; (I) 3D interaction diagram of gsa with ALOX15; (J) 3D interaction diagram of gsb with ALOX15; (K) 3D interaction diagram of gsc with ALOX15; (L) 3D interaction diagram of gsd with ALOX15; (M) 3D interaction diagram of gse with ALOX15; (N) 3D interaction diagram of gsf with ALOX15 interaction diagram; (O) 3D interaction diagram of gsg with ALOX15; (P) 3D interaction diagram of gsh with ALOX15.

Cell line toxicity prediction

In order to increase the persuasiveness of our results, we tested the theoretical inhibitory activity of the three compounds against tumor cell lines using the CLC-Pred tool [1] of the Way2Drug online server, and the results are presented in Supplementary Table 9. The online tool gives a "likelihood of activity" (P) for specific compounds. The online tool gives a "likelihood of activity" (Pa) and a "likelihood of inactivity" (Pi) for a given compound, with Pa reflecting the structural similarity of the predicted molecule to typical cell line toxicity molecules and not directly reflecting the toxicity of the compound. does not directly reflect the toxicity of the compound. Therefore, compounds with atypical structures usually have low Pa values. Previously, it has been shown that elevated ALOX15 levels are positively correlated with the development of renal cell carcinoma[2], and our prediction results also indicate that all seven candidate compounds are toxic to multiple cell lines of Renal carcinoma (Pa > Pi), with the predicted Pa values of gsa/b/c/e being more significant, which may be attributed to the fact that the parent of these derivative compounds, GS-444217 itself is a selective ATP-competitive apoptosis signal-

regulated kinase 1 (ASK1) inhibitor and has been shown in in-vivo experiments to reduce oxidative stress (OS)-induced ASK1 signalling in kidneys and inhibit acute tubular injury in rats.GS-444217 also inhibits the activation of ASK1, p38 /JNK in rat kidneys, which in turn inhibits glomerular inflammation and fibrosis [3, 4]. Seen from the other side, our present study found that GS-444217 has ALOX15 inhibitory potential, which may suggest that the mechanism of GS-444217 to suppress glomerular inflammation and fibrosis also involves preventing intracellular lipid peroxidation and free radical accumulation. i472 derivatives obtained small Pa values in the predictor, which may be attributed to the fact that the chemical structure of i472 differs from that of the typical cell line toxic structures differ greatly. Taken together, these predictions may indicate that our candidate compounds have the ability to modulate ALOX15 protein levels.

Supplementary Table 9. CLC-Pred predictions of the likelihood of Renal carcinoma cell line toxicity for seven candidate compounds.

Candidate molecules	Cell line	P(active)	P(inactive)
gsa	786-0	0.528	0.017
	TK-10	0.525	0.016
gsb	786-0	0.460	0.026
	A498	0.432	0.030
	UO-31	0.346	0.052
	SN-12C	0.270	0.086
gsc	TK-10	0.338	0.052
	786-0	0.334	0.059

	A498	0.327	0.065
	UO-31	0.287	0.083
gse	786-0	0.425	0.032
	A498	0.390	0.039
	UO-31	0.322	0.062
ia	KETR3	0.089	0.037
	786-0	0.213	0.165
ib	KETR3	0.081	0.052
ie	KETR3	0.082	0.050

Method and Material

Data set preparation and characterization

581 compounds with measured ic50 values (nmol) for ALOX15 were downloaded from the CHEMBL database and the downloaded SMILE molecular formula was converted to sdf format file using Openbabel. Among these ALOX15 inhibitory small molecules, compounds with ic50 $<1*10^3$ nmol/dm$^{\wedge 3}$ were considered to have ideal target inhibitory activity. For the sake of simplicity in calculation, their activity was noted as "1" during the ML-QSAR model training; accordingly, the remaining inactive compounds were marked as "0". The pre-processing and analysis of the molecular datasets was performed in the 2019 version of Discovery Studio software: the training and test sets were randomly divided in the ratio 7:3 (407 in training sets; 174 in test sets). Subsequently, diversity metrics were calculated for all molecules to indicate the specificity of each molecule in the data set. The metrics used to assess diversity include

ring/chain characteristics (evaluated based on Murcko assemblies), ECFP_6 fingerprint distances (whether some of the structural fragments comprising the molecule are similar), and attribute distances (physicochemical properties of the molecule) (the latter two are characterized by Tanimoto coefficients). Also, chemical spatial distributions based on four extended-connectivity/Daylight-style path-based fingerprints fingerprints fingerprints (FCFP/ECFP/FPFP/EPFP_6) were calculated for each small molecule to ensure that the molecules assigned to the training or test sets had homogeneous physical-chemical properties. Daylight fingerprints have in common that the environment around atoms is compiled into fingerprints through an iterative process, and they are generally considered to be more suitable for representing the relationships between molecular topologies and their properties due to their concise form and ability to retain as much molecular structural information as possible[5]. ECFP (Extended connectivity finger print) is obtained by compiling the environment around the atoms (atomic connection number, atomic number, atomic charge), reflecting the topological relationship between atoms in the molecule; while FCFP (Functional class finger print) is obtained by compiling the functional groups in which the atoms participate in the composition, reflecting the topological relationship between functional groups in the molecule. EPFP (Extended connectivity path-base finger print) is obtained by compiling the functional groups in which the atoms participate in the composition. Connectivity path-base finger print and FPFP (Funtional class path-base finger print), on the other hand, are obtained by analyzing all the molecular fragments in a linear path starting from one atom until reaching a specified number of bonds, and then Hasing the fragments in each path to fingerprints are generated.

Data degradation: principal component analysis

During the training of models based on large amounts of data, the resulting models or test results are often influenced by noise from irrelevant information. In order to condense and extract the independent variables that are most relevant to the numerical

characters of the data set, data degradation is applied to the data set. Principal Component Analysis (PCA) is a general method of reducing the dimensions of the data by decomposing successively the molecular properties as large as possible orthogonally onto different spatial axes (Principal Components, abbreviated as PCs), resulting in a number of PCs composed of unequal proportions to the molecular properties.

The Calculate molecule properties function of Discovery Studio was applied to calculate 55 molecular physicochemical property descriptors of ALOX15 inhibitors collected from the CHEMBL database (displayed in Supplementary Table 6), which were subsequently subjected to principal component analysis using the OPS analysis method. We excluded descriptors with low correlation with molecular activity values (reducing the number of descriptors as much as possible) to avoid over-fitting in the subsequent construction of the model: the activity correlation coefficient of each descriptor was calculated using the Pearson correlation analysis method, and those with activity correlation coefficients lower than 0.1 were excluded, while key descriptors with coefficients higher than 0.9 were retained.

Reference

1. Lagunin, A. A.-O.; Rudik, A. V.; Pogodin, P. A.-O.; Savosina, P. I.; Tarasova, O. A.-O.; Dmitriev, A. A.-O.; Ivanov, S. A.-O.; Biziukova, N. Y.; Druzhilovskiy, D. S.; Filimonov, D. A.; Poroikov, V. A.-O., CLC-Pred 2.0: A Freely Available Web Application for In Silico Prediction of Human Cell Line Cytotoxicity and Molecular Mechanisms of Action for Druglike Compounds. LID - 10.3390/ijms24021689 [doi] LID - 1689. (1422-0067 (Electronic)).
2. Gohara, A.; Eltaki N Fau - Sabry, D.; Sabry D Fau - Murtagh, D., Jr.; Murtagh D Jr Fau - Jankun, J.; Jankun J Fau - Selman, S. H.; Selman Sh Fau - Skrzypczak-Jankun, E.; Skrzypczak-Jankun, E., Human 5-, 12- and 15-lipoxygenase-1 coexist in kidney but show opposite trends and their balance changes in cancer. (1791-2431 (Electronic)).
3. Liles, J. T.; Corkey, B. K.; Notte, G. T.; Budas, G. R.; Lansdon, E. B.; Hinojosa-Kirschenbaum, F.; Badal, S. S.; Lee, M.; Schultz, B. E.; Wise, S.; Pendem, S.; Graupe, M.; Castonguay, L.; Koch, K. A.; Wong, M. H.; Papalia, G. A.; French, D. M.; Sullivan, T.; Huntzicker, E. G.; Ma, F. Y.; Nikolic-Paterson, D. J.; Altuhaifi, T.; Yang, H.; Fogo, A. B.; Breckenridge, D. G., ASK1 contributes to fibrosis and dysfunction in models of kidney disease. (1558-8238 (Electronic)).

4. Amos, L. A.; Ma, F. Y.; Tesch, G. H.; Liles, J. T.; Breckenridge, D. G.; Nikolic-Paterson, D. A.-O.; Han, Y., ASK1 inhibitor treatment suppresses p38/JNK signalling with reduced kidney inflammation and fibrosis in rat crescentic glomerulonephritis. (1582-4934 (Electronic)).
5. Rogers, D.; Hahn, M., Extended-connectivity fingerprints. (1549-960X (Electronic)).
